# Cerebral hyperactivation across the Alzheimer’s disease pathological cascade

**DOI:** 10.1093/braincomms/fcae376

**Published:** 2024-10-25

**Authors:** Nick Corriveau-Lecavalier, Jenna N Adams, Larissa Fischer, Eóin N Molloy, Anne Maass

**Affiliations:** Department of Neurology, Mayo Clinic, Rochester, Minnesota 55902, USA; Department of Psychiatry and Psychology, Mayo Clinic, Rochester, Minnesota 55902 USA; Department of Neurobiology and Behavior, University of California, Irvine 92697, CA, USA; German Center for Neurodegenerative Diseases, Magdeburg 39120, Germany; German Center for Neurodegenerative Diseases, Magdeburg 39120, Germany; Division of Nuclear Medicine, Department of Radiology & Nuclear Medicine, Faculty of Medicine, Otto von Guericke University Magdeburg, Magdeburg 39120, Germany; German Center for Neurodegenerative Diseases, Magdeburg 39120, Germany; Institute for Biology, Otto-von-Guericke University Magdeburg, Magdeburg 39120, Germany

**Keywords:** cerebral hyperactivation, Alzheimer’s disease, fMRI, amyloid, tau

## Abstract

Neuronal dysfunction in specific brain regions or across distributed brain networks is a known feature of Alzheimer’s disease. An often reported finding in the early stage of the disease is the presence of increased functional MRI (fMRI) blood oxygenation level-dependent signal under task conditions relative to cognitively normal controls, a phenomenon known as ‘hyperactivation’. However, research in the past decades yielded complex, sometimes conflicting results. The magnitude and topology of fMRI hyperactivation patterns have been found to vary across the preclinical and clinical spectrum of Alzheimer’s disease, including concomitant ‘hypoactivation’ in some cases. These incongruences are likely due to a range of factors, including the disease stage at which the cohort is examined, the brain areas or networks studied and the fMRI paradigm utilized to evoke these functional abnormalities. Additionally, a perennial question pertains to the nature of hyperactivation in the context of Alzheimer’s disease. Some propose it reflects compensatory mechanisms to sustain cognitive performance, while others suggest it is linked to the pathological disruption of a highly regulated homeostatic cycle that contributes to, or even drives, disease progression. Providing a coherent narrative for these empirical and conceptual discrepancies is paramount to develop disease models, understand the synergy between hyperactivation and the Alzheimer’s disease pathological cascade and tailor effective interventions. We first provide a comprehensive overview of functional brain changes spanning the course from normal ageing to the clinical spectrum of Alzheimer’s disease. We then highlight evidence supporting a close relationship between fMRI hyperactivation and *in vivo* markers of Alzheimer’s pathology. We primarily focus on task-based fMRI studies in humans, but also consider studies using different functional imaging techniques and animal models. We then discuss the potential mechanisms underlying hyperactivation in the context of Alzheimer’s disease and provide a testable framework bridging hyperactivation, ageing, cognition and the Alzheimer’s disease pathological cascade. We conclude with a discussion of future challenges and opportunities to advance our understanding of the fundamental disease mechanisms of Alzheimer’s disease, and the promising development of therapeutic interventions incorporating or aimed at hyperactivation and large-scale functional systems.

## Teaser

Corriveau-Lecavalier *et al*. review the emergence and progression of task-based functional MRI hyperactivation across ageing and Alzheimer’s disease, highlighting its complex relationship with Alzheimer’s pathogenesis and cognitive change. Bridging human imaging and animal studies, they provide a framework proposing hyperactivation is fundamental to the pathological cascade of Alzheimer’s disease.

## Graphical Abstract

**Graphical Abstract:**
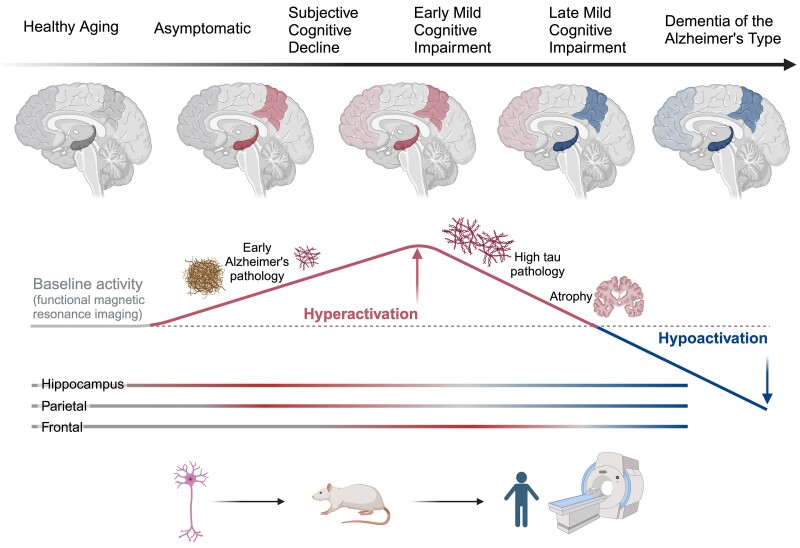

## Introduction

Alzheimer’s disease is the leading cause of degenerative dementia.^1^ Immense efforts have been deployed in the past decades to unravel the biological mechanisms involved in its progression, from the long, indolent preclinical phase to the clinical phase most commonly characterized by prominent memory problems.^2-4^ While the presence of amyloid-beta (Aβ) plaques and neurofibrillary tangles defines Alzheimer’s disease neuropathologically,^4,5^ clinical symptoms emerge from mutli-scale interactions between the accumulation of misfolded proteins and the disruption of large-scale functional systems.^6-12^ One marker of such dysfunction, called ‘hyperactivation’, has been repeatedly reported in the early stages of the disease, particularly in memory systems including the hippocampus and parietal cortex.^13-18^ In this review, we argue that hyperactivation is fundamental to the pathological cascade of Alzheimer’s disease, is closely related to cognitive symptoms and may even be a target for potential treatments.^19^

**Figure 1:**
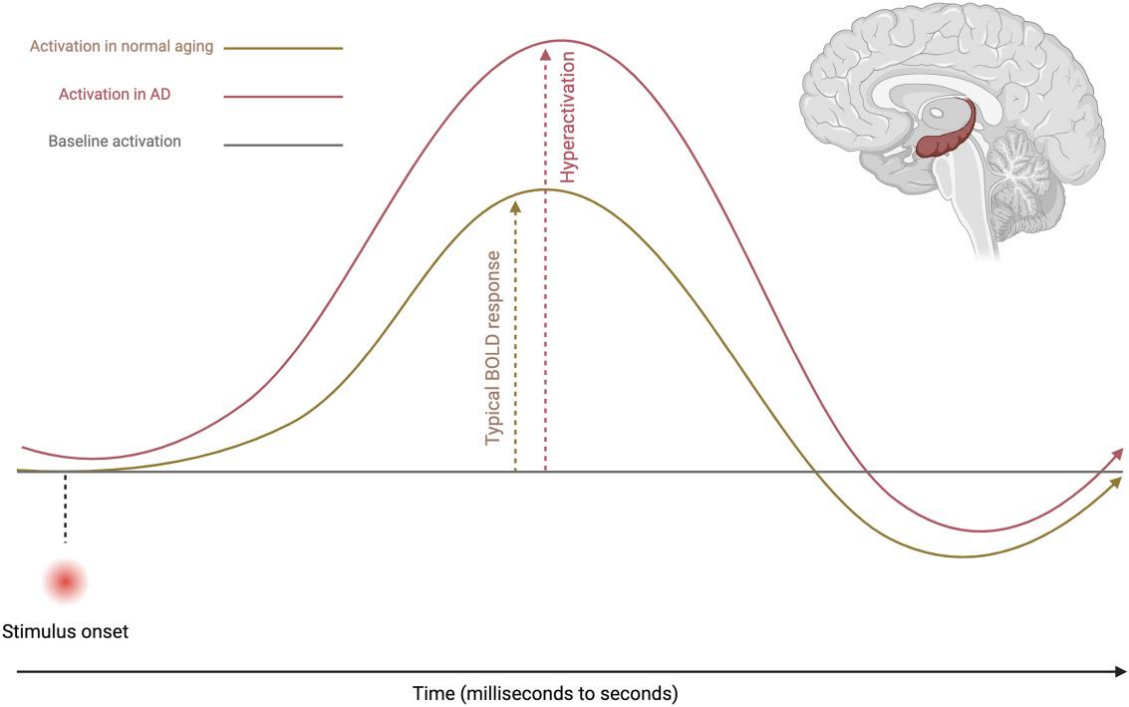
**Haemodynamic response in relation to stimulus onset during cognitively engaged states in ageing and the early stages of Alzheimer’s disease.** This is a conceptual depiction of task-related brain activation changes in the hippocampus during an episodic encoding memory task, where the solid yellow line reflects a typical BOLD signal in response to a stimulus in normal ageing. The solid red line reflects an abnormally high BOLD response in reaction to the same stimulus as seen in the early stages of Alzheimer’s disease. This is due to a range of disease-specific factors causing ‘hyperactivation’, including amyloidosis, abnormal levels of tau, neuroinflammation, etc. (see Fig. 4). Of note, baseline activation is represented by a flat line for illustration purposes only; this is not meant to accurately reflect the intrinsic fluctuations in activation/connectivity at rest/baseline.

In the context of this review, we define hyperactivation as higher blood oxygenation level-dependent (BOLD) signal on functional MRI (fMRI) in a single individual or a group of individuals either with biomarkers supportive of Alzheimer's disease (i.e. Aβ and tau) or at risk of developing Alzheimer’s disease dementia [e.g. mild cognitive impairment (MCI), *APOE4* carriership] (see Fig. 1). Moreover, we specifically refer to fMRI hyperactivation in the context of task paradigms, where both increased BOLD signal in ‘task-positive’ networks and reduced suppression of BOLD signal in ‘task-negative’ networks (or deactivation) would qualify.^20-22^ Of note, while changes in BOLD signal are observed across a wide range of neurologic and psychiatric illnesses,^23^ here we purposefully constrain the use of the term of fMRI ‘hyperactivation’ to its relation with Alzheimer’s disease. By contrast, the term ‘neuronal hyperexcitability’ refers to a cellular mechanism where neurons have an increased susceptibility to fire action potentials in response to stimuli. This term will be used only when discussing animal findings.

The earliest observations of fMRI hyperactivation were reported in the hippocampus of individuals with MCI^18,24^ or carrying an *APOE4* allele in the absence of cognitive symptoms^25,26^ while they performed in-scanner memory encoding tasks. These findings suggested that hippocampus-related memory networks become dysfunctional in early Alzheimer’s disease and that hyperactivation may help identify individuals at risk of dementia. Since these landmark studies, significant progress has been made towards elucidating the circumstances surrounding the emergence and presence of hyperactivation and its relation to Alzheimer’s disease. This is largely due to increasing efforts to detect Alzheimer’s disease in its earliest stages. Recent studies showed the presence of task-based fMRI hyperactivation prior to overt clinical symptomatology, for instance in individuals with normal cognition but presenting with subjective cognitive decline (SCD)^27-31^ and/or with *in vivo* evidence of Alzheimer’s disease pathology.^17,32-37^ The advent of PET ligands detecting Alzheimer’s pathology allowed for the assessment of close, yet complex associations between hyperactivation and the topology of Aβ plaques and tau across disease stages. Notably, early phases of Aβ-related hyperactivation followed by tau-related hypoactivation in the later stages of the disease have been documented, forming an ‘inverse U-shape’ across the disease spectrum.^38-40^ Animal models made parallel contributions by revealing a vicious, self-perpetuating cycle between Aβ, tau and disrupted neuronal circuitry.^22,41-43^ Collectively, these findings position fMRI hyperactivation as an important and early feature of Alzheimer’s disease, offering insights into the early disease stages and the intricate interplay between molecular pathology, large-scale functional systems and cognitive symptoms.

Despite these advances, a coherent narrative regarding fMRI hyperactivation and its relation to Alzheimer’s disease is still lacking. For instance, studies have found variations in the presence and spatial distribution of hyperactivation, sometimes observed alongside fMRI ‘hypoactivation’.^44,45^ These seemingly incongruent results are due to a range of factors including, but not limited to, the disease stage of the patient cohort, the specific brain areas or networks studied, the fMRI paradigm employed and the cognitive process being assessed. Moreover, the nature of hyperactivation remains a subject of debate. Some propose that hyperactivation represents a compensatory mechanism to maintain cognitive function in the context of increasing neurodegeneration.^46-48^ By contrast, others consider it as an inherently pathological phenomenon that reflects large-scale functional dyshomeostasis that contributes to, or even drives, disease progression.^6,8,14,49^

In this review, we provide a comprehensive overview of fMRI hyperactivation in the context of Alzheimer’s disease (see also Supplementary Table 1 for a detailed overview of task-based fMRI studies). Our review will commence with an examination of fMRI activation changes that occur throughout the ageing process, as well as across the clinico-pathological spectrum of Alzheimer’s disease. We will then discuss evidence supporting the close relationship between hyperactivation and *in vivo* markers of Alzheimer’s pathology. This will primarily be supported by task-based fMRI studies, but will also draw from studies using different functional imaging techniques and animal models. We will then delve into the potential mechanisms underlying hyperactivation within the context of Alzheimer’s disease, and a consideration of the evidence suggesting hyperactivation is a maladaptive rather than a compensatory process. Finally, we offer an operational framework that bridges hyperactivation, ageing, cognition and the Alzheimer’s disease pathological cascade.

Our focus will be centred around the canonical, amnestic variant of the disease and its prodromal phase, given that hyperactivation has almost exclusively been studied in this phenotype. Consequently, a large proportion of studies discussed throughout the review draw on memory-based paradigms and functional systems supporting this mental function. However, when available, we also cite newer studies suggesting that the phenomenon of hyperactivation may extend to non-memory systems and atypical variants of Alzheimer’s disease. We conclude by discussing the future challenges and opportunities that lie ahead in advancing our comprehension of the fundamental disease mechanisms of Alzheimer’s disease, with a particular focus on the development of therapeutic interventions incorporating or aimed at hyperactivation and large-scale functional systems.

## Age-related differences in fMRI activation

### Functional changes to the medial temporal lobe and hippocampal circuit

Ageing is associated with many changes to the brain, including neurodegeneration, synaptic loss, decreases in white matter integrity and altered metabolism.^50-53^ One region that is particularly vulnerable to these effects is the medial temporal lobe (MTL). The MTL encompasses structures such as the hippocampus, amygdala, entorhinal cortex, perirhinal cortex and parahippocampal cortex and serves as a critical region to support memory processing.^54^ The MTL also appears to be one of the first regions to demonstrate changes in fMRI activation during the ageing process.

The wiring of the hippocampal circuit, which begins with input from the entorhinal cortex to the dentate gyrus and CA3 hippocampal subfields via the perforant pathway,^55^ renders the hippocampus uniquely prone to hyperactivation if normal input becomes disrupted.^56^ The structural integrity of the perforant pathway has been shown to be reduced within both aged rodent models^57^ and healthy older adults (OAs) using diffusion MRI.^58-60^ It has been hypothesized that without proper input from the entorhinal cortex and dentate gyrus, the recurrent collaterals within CA3 become disinhibited,^56^ which leads to unconstrained activation of these auto-associative connections that may express as increased activation during fMRI tasks. Further, inhibitory interneurons within the hippocampus have been found to be particularly impacted by ageing,^61,62^ potentially shifting the excitatory–inhibitory balance in favour of over-excitation.^63^ While these changes are not as dramatic as the widespread neuronal loss associated with disease, they impact the functional balance of the MTL, leading to changes in relative input and processing loads across these highly connected regions.

**Figure 2:**
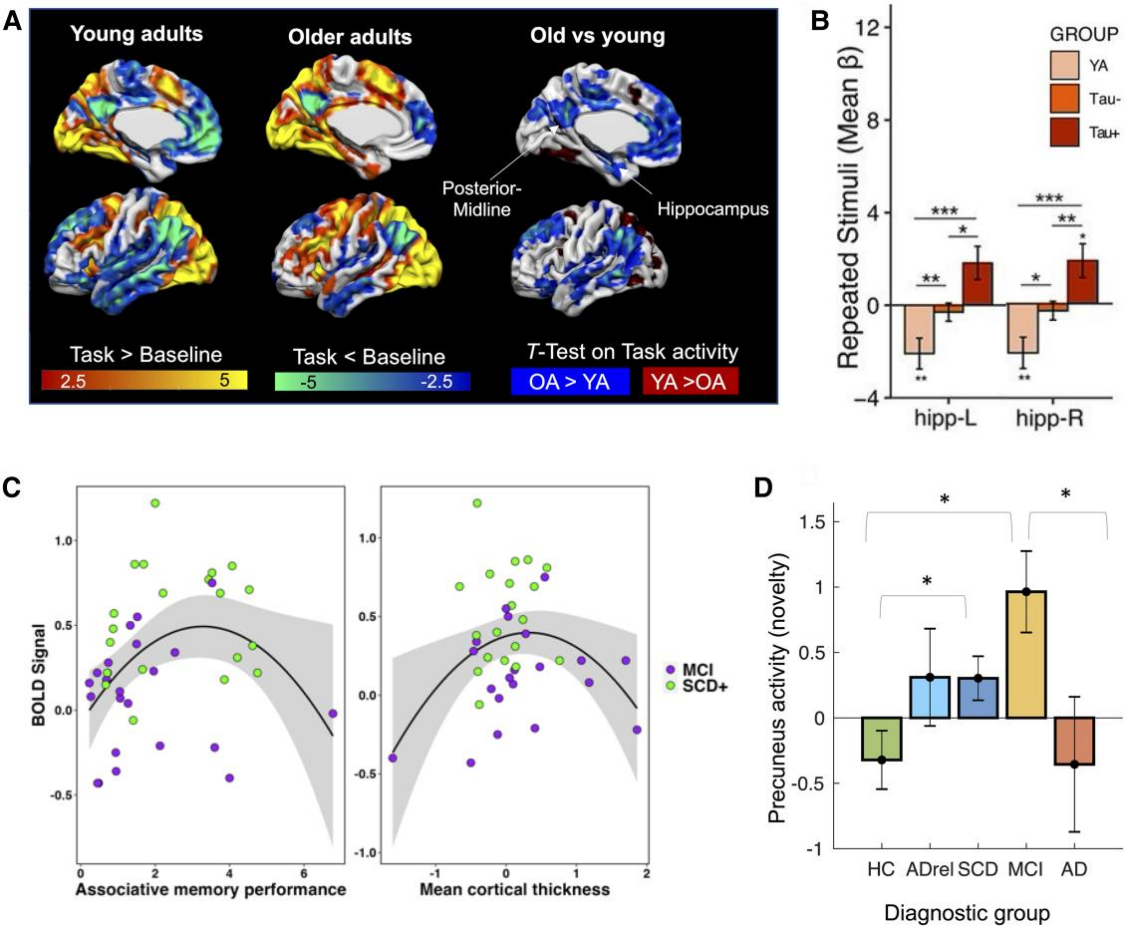
**Task-related activity alterations in ageing and clinical at-risk groups for Alzheimer’s disease.** (**A**) Young adults (YA; *N* = 23) show increased fMRI activity during a memory task relative to a perceptual baseline mainly in visual and temporal areas (yellow), whereas decreased activity is seen in the default mode network including posterior-midline and parietal regions (blue). OAs (*N* = 49) show a similar pattern (*middle*), but deactivations are significantly reduced during the task relative to YAs as further shown by a two-sample *t*-test (*right*; blue areas display areas of increased activity in OAs). The results are shown with *P*-voxel < 0.005 and *P*-cluster < 0.05 (family-wise error [FWE]-corrected). Data re-analysed from Maass *et al*.^17^  **(B)** During a memory task, hippocampal (‘hipp.’) activity (i.e. reduced deactivation) for repeated stimuli was increased in tau-negative OAs (*N* = 29) compared with YAs (*N* = 21), and this increased activity was further exacerbated in the presence of tau (tau-positive OAs; *N* = 16; repeated measures ANOVA; ***P < 0.001, **P < 0.01, *P < 0.05). Figure adapted from Adams *et al*.^36^ (originally published under the terms of Creative Commons Attribution 4.0 licence). **(C)** Corriveau-Lecavalier *et al*.^27^ found a quadratic (inverted U-shape) relationship between proxies of disease severity and left superior parietal task-fMRI activity in a group of patients with SCD (*n* = 54) and MCI (*n* = 26) using quadratic regression models (*F* = 3.773, *P < 0.01 for associative memory and *F* = 5.303, *P < 0.05 for cortical thickness). Data replotted from Corriveau-Lecavalier *et al*.^27^  **(D)** Precuneus fMRI activity during novelty processing followed an inverted U-shape pattern across diagnostic groups (one-way ANOVA, *F*(3472) = 4.31, *P = 0.005) with increasing Alzheimer’s disease risk, with increased activity in subjective cognitive decline (SCD; *N* = 222) and MCI (*N* = 82) that is reduced in Alzheimer’s disease dementia (*N* = 32). *denotes significant group differences (*post hoc* tests) surviving Bonferroni–Holm correction with *P* < 0.05. ADrel, first-degree relatives of patients with dementia; HC, cognitively and subjectively healthy controls (*N* = 163). Data taken and replotted from Billette* *et al*.^31^.

Consistent with these models,^63^ several fMRI studies have reported increased fMRI activation in the hippocampus in OAs (see example in Fig. 2A and B). Due to the MTL’s critical role in learning and memory, the majority of previous studies have probed how fMRI activation is altered in the context of tasks taxing various memory processes. For example, hippocampal activation, and particularly within the dentate gyrus/CA3 subfields, has been shown to be higher in cognitively normal OAs compared with young adults during mnemonic discrimination tasks that tax pattern separation, a computation supporting orthogonalization of distinct memories performed within the dentate gyrus and CA3.^64-66^ Furthermore, a recent quantitative meta-analysis^67^ aggregated 45 fMRI studies of autobiographical memory retrieval and found overall higher bilateral hippocampal fMRI activation (as well as in the precuneus/retrosplenial cortex and temporal cortex) in OAs relative to young adults, supporting age-related differences in recruitment of the hippocampus during retrieval.

Age-related activation changes in older compared with younger adults have also been observed with a variety of other task paradigms spanning many MTL regions.^64,68-71^ For example, a study by Berron *et al*.^70^ showed that regions such as the anterolateral entorhinal cortex and perirhinal cortex demonstrated reduced domain-specific activation patterns for object versus scene memory. This loss of domain specificity has been also interpreted as increased similarity in functional responses across different tasks in ageing, a phenomenon also known as ‘dedifferentiation’. Similar findings of dedifferentiation were observed during successful memory encoding in the parahippocampal cortex for scenes versus objects, and this was associated with worse item memory.^72^ Additionally, a study by Reagh *et al*.^64^ demonstrated that while the dentate gyrus and CA3 have increased activity during object pattern separation compared with young adults, the anterolateral entorhinal cortex has reduced activity, suggesting a functional imbalance within the MTL. Furthermore, Ankudowich *et al*.^71^ found widespread and variable patterns of increased brain activation in OAs that differed on the basis of encoding and retrieval. Activation in the fusiform cortex increased with age during both encoding and retrieval, while activation in the hippocampus increased with age during the retrieval phase. These age-related increases in hippocampal activation predicted worse retrieval accuracy, suggesting an age and performance trade-off.

Finally, we note that several studies have instead found an age-related reduction in BOLD signal in the MTL. For instance, Salami *et al*.^73^ reported encoding-related activity reduction in the bilateral hippocampus in a large population-based ageing sample during a face–name memory task. In the same cohort, Pudas *et al*.^74^ found that during encoding, OAs with stable cognition had similar hippocampal activity relative to young adults, whereas hippocampal activity was lower in OAs whose memory declined. Together, these studies suggest that activation differences associated with ageing occur throughout the MTL and in the context of many cognitive processes, where the pattern of age-related activity changes may depend on the specific task, contrast and performance level. This emphasizes the potential role of activation changes contributing to age-related variability in memory performance.

A major caveat to the interpretation of previous studies of ‘normal’ (non-pathological) ageing is that the majority of studies did not have Alzheimer’s pathology biomarker status available. This precludes the ability to confirm that these participants were not in the preclinical stage of Alzheimer’s disease (i.e. positive for Aβ and tau pathologies) or free from pathology found in other neurodegenerative diseases (e.g. a-synuclein and TDP-43). With the increased availability of PET and CSF biomarkers, and more recently, plasma-based biomarkers,^75^ staging can now be incorporated to confirm the absence of Alzheimer’s disease pathology. Recent evidence incorporating Alzheimer’s biomarkers supports that increased fMRI activation may emerge prior to the prodromal phase of Alzheimer’s disease. For example, Adams *et al*.^36^ showed that tau-PET-negative OAs had increased hippocampal activation during the repeated stimuli presentation of a mnemonic discrimination task compared with young adults (Fig. 2B). Interestingly, this response was further increased in tau-PET-positive OAs, supporting the hypothesis that functional activation becomes exaggerated in the very early stages of Alzheimer’s disease (further discussed in subsequent sections).

### Functional changes to parietal and posterior-midline regions

Increased task-based fMRI activation in cognitively normal OAs relative to young adults has been also reported in posterior-midline and parietal regions.^76-78^ Posterior-midline and parietal regions including the precuneus, posterior cingulate, retrosplenial cortex and lateral parietal cortex typically demonstrate reduced fMRI activation during initial encoding of novel information, also referred to as task-related ‘deactivation’^79-82^ as shown in Fig. 2A. These brain regions, together with medial prefrontal regions, form the ‘default mode network’ (DMN). The DMN is usually suppressed during external tasks demands but is active in situations requiring remembering (repetition), focusing on internally represented information, envisioning the future and making social inferences.^83,84^ It is of note that the definition of the DMN slightly varies across parcellations, where some include the hippocampus while others do not.^85-87^ In the context of this review, we discuss functional alterations in the DMN and hippocampus separately for several reasons. With respect to episodic memory, deactivation of the DMN is thought to reflect the proper reallocation of neuronal resources necessary for successful encoding,^88,89^ while increased activation of the hippocampus supports the encoding of novel information but is suppressed during stimulus repetition.^36,79,90^ Moreover, the DMN and hippocampus are differentially related to Alzheimer’s disease pathology, whereby the former initially accumulates Aβ before being targeted by tau and the latter is far more susceptible to tau pathology.^6,8,17,91^

In an early landmark fMRI study, Lustig *et al*. employed an alternating block design with active semantic classification and visual fixation to assess patterns of fMRI activation in older relative to younger adults.^77^ They found that task-related deactivation in medial frontal regions and posteromedial cortex was reduced in older participants, where these regions initially activated in all groups, but quickly deactivated relative to fixation only in young adults. Similarly, Vaninni *et al*.^78^ showed that the posteromedial cortex is deactivated during initial encoding of face–name pairs and this deactivation decreases with repetitive encoding (‘repetition enhancement’) in young adults. However, OAs had less deactivation during first stimulus encoding and a diminished stepwise change in deactivation with repeated encoding compared with younger adults. Together, these early studies show reduced deactivation and modulation of posterior-midline regions when OAs are engaged in a task (see also Fig. 2A). A recent study^92^ in OAs further found that DMN midline structures not only deactivate less during successful encoding of novel scenes, but also show reduced resting-state BOLD amplitude fluctuations, indicating lower modulation of the BOLD signal in DMN regions even at rest. Further, higher encoding-related activity in the precuneus was related to worse memory performance across older participants,^76,93^ suggesting that an imbalance between task-positive and task-negative networks may be detrimental.

### Functional changes to frontal regions

Frontal areas are involved in a wide range of cognitive functions, including executive functions, attentional capacities and complex problem-solving. Studies in ageing have mostly reported increased task-based fMRI activation in prefrontal areas relative to younger adults,^73,89,94-98^ which was often found when cognitive performance was maintained over time or similar to younger counterparts. Interestingly, this pattern has also been observed in MCI.^99^ Collectively, these findings have led to the hypothesis that increased frontal activation may reflect enhanced top-down cognitive control in response to greater attentional demands.^89,98^ It is, however, important to note that increased prefrontal activity has also been interpreted as reduced efficiency in processing.^100-103^ For instance, a recent study using multivariate Bayes analysis showed that frontal activation did not carry additional information beyond that provided by posterior regions during a visual memory task.^102^ This finding questions the compensatory role of increased prefrontal activation in normal ageing, although further confirmation by independent studies is required.

### Interactions and vulnerabilities between systems

The MTL and parietal lobe have strong bidirectional anatomical connectivity^104^ and form a highly interactive memory system.^105,106^ Thus, age-related activation changes in one region may disrupt the functional balance of the entire system. Supporting this hypothesis are studies in OAs that show alterations in MTL-parietal functional connectivity both at rest and during task.^107-110^ Moreover, preserved intrinsic connectivity between the hippocampus and posteromedial cortex in ageing has been associated with better memory performance.^111,112^ A study using dynamic causal modelling (DCM) to investigate activation during successful encoding of novel scenes demonstrated that OAs exhibited attenuated inhibitory parahippocampal cortex–precuneus connectivity compared with younger adults, and this pattern was associated with worse memory performance.^108^ Diersch *et al*.^69^ additionally showed reduced inhibitory self-connection strength (i.e. relative ‘disinhibition’) in the anterior hippocampus by means of DCM, as well as aberrant learning-related dynamics in the parietal lobe compared with young adults. Together, these findings point towards reduced inhibition within the hippocampus as well as reduced suppression of information flow from the MTL to the posterior-midline regions in ageing. Further, a disconnection of the MTL from the parietal lobe has been proposed to lead to unconstrained hippocampal activation^113-116^ (see Pasquini *et al*.^117^ for a review). However, it is still unclear whether MTL and parietal activation changes begin simultaneously, or whether dysfunction within one region initiates a chain of functional alterations that disrupts activation across the system. Early age-related alterations in hippocampal circuitry^118,119^ suggest that dentate gyrus/CA3 activation changes may precede changes to parietal activation. However, this proposed temporal cascade has not yet been directly assessed with fMRI.

The age-related changes in functional activation within and between the MTL and parietal lobe may render these regions to be selectively vulnerable to Alzheimer’s disease.^120,121^ Research spanning across many different neurodegenerative diseases has suggested that the functional and structural vulnerability of regions throughout the lifespan may predispose to disease effects.^122-124^ As the ageing process leads to regional functional dysregulation, this disruption of normal homeostatic mechanisms may confer an inherent vulnerability and/or lack of resistance to the accumulation of pathological proteins. In sporadic Alzheimer’s disease, for which the greatest risk factor is age, heightened levels of activation as a result of the ageing process may trigger a large-scale functional dyshomeostasis associated with the increased production of pathological proteins.^125^ The following sections describe how these functional abnormalities manifest across the clinical spectrum of Alzheimer’s disease with a focus on individuals with SCD, MCI and Alzheimer’s disease dementia (see also^126,127^ for fMRI meta-analyses).

## Hyperactivation in the clinical spectrum of Alzheimer’s disease

### Alzheimer’s disease dementia

Early studies using fMRI to probe patterns of brain activation in Alzheimer’s disease have largely focused on patients with clinically defined dementia and relied on task paradigms targeting episodic memory. The majority of these studies have reported lower fMRI activation in patients with dementia compared with cognitively healthy controls, a phenomenon known as ‘hypoactivation’.^128-136^ This hypoactivation was observed in the hippocampus, the MTL and temporo-parietal regions during associative memory (i.e. face–name association)^128,133,134^ or visual encoding (e.g. of scene images).^129,132^ Hypoactivation was generally interpreted as an inability to activate memory-related brain areas to an extent that is similar to healthy controls, leading to poor memory performance. Rarely have studies also assessed non-cognitive domains in Alzheimer’s dementia. A study by Wright *et al*.^137^ found hyperactivation in the amygdala in patients with dementia compared with elderly and young controls when viewing faces. Importantly, the level of activation in these patients correlated with the severity of affective symptoms, suggesting that patterns of hyper- versus hypoactivation may also track with non-cognitive symptoms.

### Mild cognitive impairment

In parallel, studies conducted in individuals with MCI reported paradoxical fMRI hyperactivation compared with cognitively normal counterparts. The presence of hyperactivation was first documented by Dickerson *et al*.,^18^ where higher fMRI BOLD signal was observed in the hippocampus in individuals with amnestic MCI, while they performed a visual memory task. Interestingly, those who exhibited the highest levels of activation also showed more rapid cognitive decline over a 30-month follow-up. This initial finding suggested that fMRI hyperactivation represents an early functional signature of Alzheimer’s disease and may herald progression to dementia. Subsequent studies reported similar findings in patients with MCI, demonstrating hippocampal^14,24,138-143^ as well as prefrontal and parietal^15,16,99,126,127,144,145^ hyperactivation, while participants performed various episodic and working memory tasks. It is, however, essential to mention that other studies reported the opposite pattern in patients with MCI, with hypoactivation in the hippocampus,^146,147^ lateral entorhinal cortex,^148^ prefrontal cortex^149^ and posterior cingulate^150^ during verbal encoding memory tasks, which is reminiscent of patterns reported in Alzheimer’s disease dementia.

In an attempt to reconcile the seemingly contradictory results in MCI, a hypothesis was put forth that the observed patterns of activation depend on clinical severity. A study by Celone *et al*.^128^ specifically tested this hypothesis by comparing ‘early’ and ‘late’ MCI (based on a clinical scale) to cognitively healthy controls and patients with Alzheimer’s disease dementia during a face–name associative memory task. Compared with healthy controls, the early MCI group showed hippocampal hyperactivation, while both late MCI and dementia groups showed hypoactivation. These results were replicated by independent studies,^99,144^ supporting that clinical severity is tied to hyper- or hypoactivation.

Other studies assessed the presence of fMRI hyperactivation in MCI as a function of specific cognitive contrasts. For instance, Johnson *et al*.^151^ reported reduced hippocampal repetition suppression due to increased activity for familiar faces in MCI. Thus, hyperactivation in MCI or mild Alzheimer’s disease dementia may manifest as reduced suppression to familiar/repeated stimuli as supported by studies assessing novelty-related activity in the MTL.^31,36^ Another study by Clément and Belleville^144^ found that associative memory paradigms elicit hyperactivation in early but not late MCI, whereas paradigms involving item memory elicit hyperactivation in late but not early MCI. This suggests that the topology of hyperactivation is sensitive to the disease stage at which the network subserving the cognitive process is affected. For instance, paradigms tapping into associative memory and mnemonic discrimination are more likely to elicit hyperactivation in the early disease phase, whereas those tapping into item memory or executive functioning^99^ are expected to provoke hyperactivation in the later disease stages.

### Preclinical stages of Alzheimer’s disease and APOE4 carriers

An increased interest in the early identification of Alzheimer’s disease led to the investigation of the presence of hyperactivation in individuals at risk of Alzheimer’s dementia. For example, studies have reported hippocampal and cortical hyperactivation in individuals with SCD (Fig. 2C and D), while they performed various associative and item novelty memory tasks.^27-29,31,152^ As noted earlier, studies have also revealed higher fMRI activation in younger and cognitively normal OAs carrying an *APOE4* allele^25,26,153-156^ This suggests that hyperactivation may antedate the onset of memory complaint in the setting of developmental factors predisposing to developing Alzheimer’s disease. These findings echo studies reporting abnormal functional connectivity patterns within the hippocampus and the DMN in individuals with SCD^28,157^ or carrying an *APOE4* allele^158,159^ and that focal hyperactivation may reflect large-scale functional abnormalities within memory networks.^28^

Altogether, findings across the clinical spectrum of Alzheimer’s disease are highly indicative of task-based hyperactivation as an early feature of the disease, followed by hypoactivation in the later stages, forming a non-linear ‘inverse U-shape’ across the disease spectrum.^27,160^ However, a major caveat of most studies described above is the lack of *in vivo* biomarkers of Alzheimer’s disease pathology. Tying these activation dynamics to Alzheimer’s disease pathology is therefore critical to understanding how hyperactivation contributes to the pathological cascade and cognitive symptoms of the disease.

## Associations between hyperactivation and Alzheimer’s disease pathology

### FMRI hyperactivation is related to biomarkers of Aβ or tau pathology

**Figure 3:**
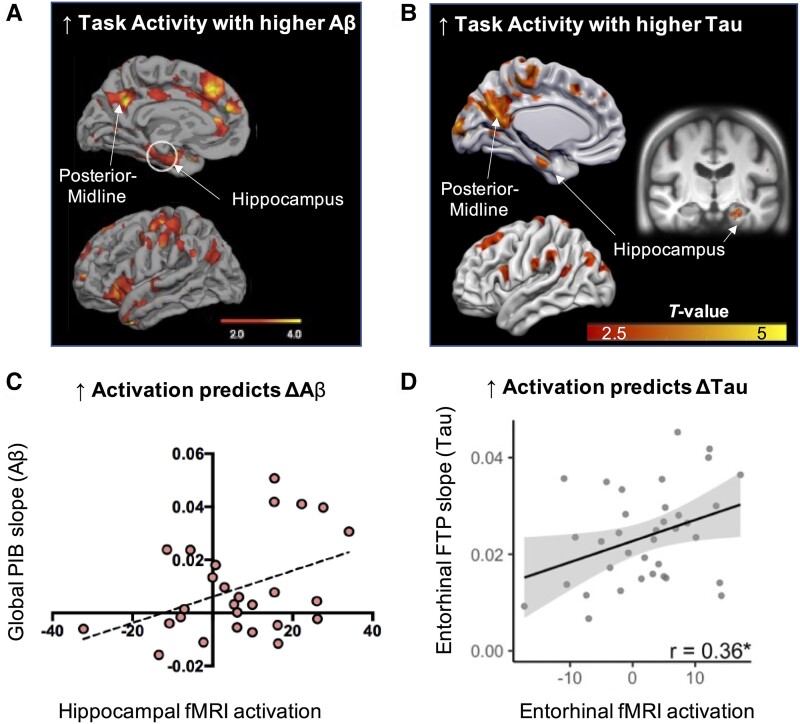
**Associations between increased fMRI task activity and Alzheimer’s disease pathology in cognitively normal OAs.** Increased task-related activity has been related to increased Aβ (**A**; *N* = 35) and tau (**B**; *N* = 49) pathology in cognitively unimpaired individuals. Regions that show increased task activity in relationship with early Alzheimer’s disease pathology include the MTL (hippocampus) and posterior-midline (precuneus, posterior cingulate). (**A**) Reprinted with permission from Sperling *et al*.^21^ (**B**) Data taken from Maass *et al*.^17^ The results are FWE-corrected at cluster level (*P*-voxel < 0.005 and *P*-cluster < 0.05). Longitudinal PET studies have found that higher task activity in the MTL at baseline predicts increased accumulation of Aβ [**C**; *N* = 27; linear mixed-effects model on Pittsburgh Compound-B (PIB): time × activity interaction; *t*(12) = 3.58, *P* = 0.004] and tau (**D**; *N* = 37; Pearson correlation: *r* = 0.36, *P < 0.05), both measured with PET, over time. Reproduced from (**C**) Leal *et al*.^33^ and (**D**) Adams *et al.*^163^ (originally published under the terms of Creative Commons Attribution 4.0 licence). FTP, flortaucipir.

Early studies examining the relationship between Alzheimer’s disease pathology and fMRI activation focused on the effects of Aβ owing to the earlier development of PET ligands targeting this pathology (e.g. ^11^C-Pittsburgh Compound-B, ^18^F-florbetapir). These studies revealed that Aβ pathology is related to increased fMRI activation. For example, Aβ-positive cognitively normal OAs show reduced entorhinal functional deactivation^119,161^ and increased functional connectivity in circuits associated with the entorhinal cortex,^119,161^ alterations that go beyond that of typical age-related reduced deactivation. Other studies reported complementary findings of reduced deactivation of task-negative regions in older Aβ-positive participants^21,32,93,162^ with increased fMRI activation specifically in the precuneus and posterior cingulate cortex (see Fig. 3A).^21^ Furthermore, Aβ-related hyperactivation in frontoparietal control regions has been reported during working memory in cognitively normal participants.^164^ Similarly, fMRI hyperactivation has been observed in the hippocampus of Aβ-positive relative to Aβ-negative patients with MCI.^165^ This increase in activation was found both cross-sectionally and longitudinally, with higher hippocampal activity at baseline and over a 3-year timespan, despite reduced hippocampal volume and increasing cognitive decline over time.^165^ This body of evidence indicates a link between Aβ accumulation and both fMRI hyperactivity and functional connectivity of the hippocampal formation, MTL cortex and regions within the DMN.

The more recent emergence of PET tracers targeting hyperphosphorylated, aggregated tau pathology such as ^18^F-flortaucipir has enabled critical examinations of the relationship between fMRI activation and tau pathology (see Fig. 3B). One consistent finding across studies is the association between tau deposition and hyperactivation within the hippocampus in cognitively normal participants. Increased hippocampal activation across a variety of memory domains, such as mnemonic discrimination^17,34,36,66^ and successful encoding,^35^ has been shown to correlate with higher tau-PET deposition within the medial and inferior temporal lobe.^35,36,66^ This close association likely results from the entorhinal cortex’s early predisposition to developing tau pathology,^125^ which may affect normal processing within the hippocampus, leading to hyperactivation within its recurrent circuitry.

Fluid biomarkers of phosphorylated tau (p-tau) that measure release of soluble tau species, such as p-tau181, p-tau231 and p-tau217 derived from CSF or plasma, have also been investigated in the context of fMRI activation changes. For example, previous work has found that higher CSF p-tau181 is associated with hyperactivation in attentional control regions (i.e. parieto-frontal) during two different attention tasks^37^ and with hyperactivation in the hippocampus during mnemonic discrimination.^34^ These results support the general association between Alzheimer’s disease neuropathological change and hyperactivation, as current p-tau biomarkers may reflect both Aβ and non-aggregated forms of phosphorylated tau, particularly in cognitively normal populations.^166^

### Consistency of fMRI hyperactivation with Aβ and tau pathology

Evidence pointing to whether fMRI activation is more closely related to tau or Aβ pathology, or even their interaction, is less consistent. Regardless, the regional pattern of increased fMRI activation is strikingly similar (see Fig. 3A and B). While some studies demonstrate associations between both pathologies and fMRI activation changes, the specific contrasts associated with each differ,^66^ or the direction of activation changes are opposing.^35^ For example, in a study by Marks *et al*.^66^ in which cognitively normal OAs performed an object mnemonic discrimination task, tau and Aβ each showed associations with activation in the MTL defined by different task contrasts. Specifically, hippocampal and entorhinal tau was associated with increased activation during encoding for subsequent false alarm stimuli, while global Aβ was associated with reduced deactivation during encoding for subsequent hit stimuli. These discrepant findings highlight the need for more studies to fully characterize how tau and Aβ may map onto distinct or overlapping aspects of hyperactivation.

A number of recent studies have demonstrated tau-related hyperactivation while not supporting an association between Aβ and fMRI activation changes.^17,36,37,70^ For example, tau-positive cognitively normal OAs showed activation increases during object–scene processing in cortical regions^17^ and during repeated stimuli presentations in MTL^36^ compared with tau-negative OAs. However, these activation differences were weaker when comparing Aβ-positive with Aβ-negative participants. Additionally, studies assessing activation with CSF measures of pathology found associations with p-tau181, but not Aβ42/40^34,37^ Furthermore, while Huijbers *et al*.^35^ did not find a direct relationship between Aβ and activation, global Aβ exhibited a negative association with activation when entered together into a model with inferior temporal tau, opposing the tau-related increased activation. While these results are more difficult to reconcile with initial findings indicating Aβ-activation relationships, this apparent lack of evidence indicating a relationship between hyperactivation and Aβ may be a result of methodological differences across studies, or insufficient power to capture Aβ-related activation effects. In this respect, soluble Aβ oligomers may be the key drivers of neuronal hyperactivity^167^; however, current Aβ-PET tracers only measure aggregated Aβ plaques, which may not capture critical Aβ-activation relationships in humans.^168^ Further, the general focus on MTL activation in the context of tau may preclude discovery of other activation patterns more closely related to Aβ, such as decreased deactivations in posterior-midline regions where Aβ primarily aggregates.

Finally, converging evidence from resting-state, rather than task-based, fMRI studies further demonstrate the role of tau and Aβ pathology in regional and whole-brain network disruptions,^38,119,169-171^ These studies largely converge and suggest that Aβ may be associated with initial increases in functional connectivity, perhaps reflecting coordinated and aberrant activation. In contrast, increasing levels of neocortical tau pathology that emerge later in disease progression are associated with reduced connectivity strength. Although these findings are harder to interpret in the context of ‘hyperactivation’ as there is no baseline for statistical comparison, findings from resting-state fMRI are consistent with regionally specific findings of increased task-based fMRI activation. Furthermore, they provide insight as to how large-scale networks may be impacted by local activation changes,^92^ reflecting the interconnected nature of distinct brain regions and vulnerability of large-scale systems to pathology.

### FMRI hyperactivation relates to longitudinal accumulation of Alzheimer’s disease pathology

A compelling open question in the aetiology of Alzheimer’s disease is the directionality between the emergence of hyperactivation and the development of Alzheimer’s pathology, particularly in the early, pre-symptomatic stages of Alzheimer’s disease. Inspired by studies in animal and *ex vivo* models demonstrating increased neuronal activation leads to greater production of pathological proteins^172-174^ (further reviewed in a subsequent section), human neuroimaging studies have attempted to generate evidence to answer this critical question.

Neuroimaging data in humans point to patterns of increased fMRI activation preceding the development of Aβ pathology. For example, in a longitudinal study by Leal *et al*.,^33^ higher baseline hippocampal activity assessed during a memory encoding task was associated with an increased rate of global Aβ-PET accumulation across cognitively normal OAs (Fig. 3C), which was paralleled by cognitive decline. Supporting this, working models have suggested that increased activation over the lifespan may leave certain regions predisposed to Aβ accumulation,^175^ which is supported by hyperactivation found in *APOE4* carriers that persists from mid-life onwards,^153,154,176^ and the propensity of Aβ to accumulate in metabolically active ‘hub’ regions.^177^

Recent longitudinal studies have also demonstrated that increased fMRI activation predicts the accumulation of tau pathology within the MTL.^91,163^ In a study by Adams *et al*.,^163^ increased fMRI activation at baseline in the entorhinal cortex and parahippocampal cortex was associated with longitudinal increases in tau-PET accumulation in the respective regions (Fig. 3D). Furthermore, higher baseline hippocampal activation was associated with longitudinal increases in tau accumulation specifically in the entorhinal cortex, suggesting that tau accumulation and hyperactivation within the MTL circuit may be linked. A recent study by Giorgio *et al*.^91^ extended this work by using DCM to demonstrate that Aβ-related ‘hyperexcitability’ of the DMN led to MTL network ‘hyperexcitability’, which subsequently predicted tau accumulation in the entorhinal cortex. This finding suggests that hyperactivity of distant regions, perhaps emerging in part due to development of Aβ, is associated with widespread network changes that may contribute to tau accumulation. Finally, patterns of functional connectivity from resting-state fMRI strongly predict the spatial pattern and the accumulation rate of tau pathology,^178-180^ suggesting that the combination of structural projections^181,182^ and activation may partly underlie observed patterns of tau pathology.

Overall, initial neuroimaging evidence in human samples supports the hypothesis of activity-dependent Aβ and tau production, particularly in the early stages of Alzheimer’s disease. However, it is important to note that additional factors not considered in these previous studies may contribute to both the development of pathology and fMRI hyperactivity, precluding the interpretation of a directional mechanistic link. Regardless of the initiating factor, hyperactivity and pathology, and in particular Aβ, may act upon each other in a vicious cycle, causing increasingly higher levels of each. While animal model evidence closely points to hyperexcitability leading to tau pathology, the reverse is not as clearly substantiated, with tau, in fact, being shown to lead to neuronal silencing and hypoactivation in animal models^43^ (but see also^183^). The neurobiological mechanisms linking hyperexcitability to pathology, providing an explanatory account of possible factors leading to task-based fMRI hyperactivation, are further reviewed in the following section.

## Underlying mechanisms and implications of hyperactivation

### Hyperactivation–pathology relationships in animal models

Animal studies designed to recapitulate the pathological hallmarks of Alzheimer’s disease strongly suggest that neuronal hyperactivity is a causal factor for ageing and Alzheimer’s disease-related memory deficits (for review, see e.g.^19,184,185^). In pathology-free aged rodents^186,187^ and monkeys^188^ with memory impairment, hyperactive neurons with elevated firing rates have been localized in the CA3 subfield of the hippocampus (particularly in proximal CA3^189^), but also in connected posterior cortex.^190^ Several mechanisms may be involved in driving hippocampal hyperactivity in ageing, including altered input of entorhinal cortex to dentate gyrus (DG) and CA3 via the perforant path,^191-193^ redistribution of synaptic weights in CA3,^194^ reduced cholinergic modulation of CA3 interneurons by the medial septum^195^ and decreased interneuron activity.^188,196^

Increased activity in CA3 neurons in aged memory-impaired animals is thought to impair computational properties of the hippocampus, with a shift from pattern separation towards pattern completion manifesting as a behavioural impairment in the ability to discriminate between similar stimuli.^19,63,197^ These findings of CA3 hyperactivity in aged animals are congruent with fMRI studies in OAs that similarly localized increased activation during mnemonic discrimination task to the dentate gyrus and CA3.^64-66^ Notably, low-dose administration of the antiepileptic levetiracetam^187^ reduced hyperactivity in CA3 and posterior cortical regions of aged rodents, which aligns with the posterior components of the DMN in humans.^19^ Further, treatment with levetiracetam as well as with selective GABA-Aα5-positive allosteric modulators was linked to improved memory performance in aged rats.^190,198,199^

Transgenic Alzheimer’s disease mouse models have further revealed a causal link between neuronal hyperactivity/hyperexcitability and the progression of Aβ and tau pathology.^19,184,185^ In mice overexpressing human Aβ, hyperexcitable neurons co-localize with Aβ plaques^200,201^ and administration of Aβ oligomers can also induce neuronal hyperexcitability in the hippocampus and cortex.^167,202^ Early hyperactivity in the lateral entorhinal cortex has been further associated with elevated levels of Aβ precursor protein metabolites in a transgenic mouse model of Alzheimer’s disease.^203^ At the synaptic level, hyperactivity induced by Aβ oligomers has been related to a dysfunctional reuptake of extracellular glutamate^41^ and disruption of homeostatic synaptic plasticity mechanisms that normally maintain a setpoint of activity.^204^ Furthermore, neuronal hyperexcitability can also increase production and release of Aβ and tau^20,173,174,205^ and enhance the spread of tau pathology in the hippocampus and associated circuits.^172^ Interestingly, neuronal hyperexcitability in mouse models of Alzheimer’s disease can be rescued by β- and γ-secretase inhibition to reduce soluble Aβ levels,^167,202^ and in turn, activity attenuation can reduce both Aβ aggregation^206^ and learning and memory deficits.^207^

Finally, mouse models expressing *APOE4* have further revealed that GABAergic interneurons in the MTL are specifically susceptible to *APOE4*-mediated toxicity. Mice that express the human *APOE3* or *APOE4* gene displayed hyperactivity in the entorhinal cortex that was driven by decreased inhibition.^208^  *APOE4*-knockin mouse models also showed an age- and tau-dependent decrease in hilar GABAergic interneurons in the hippocampus, which can lead to inhibitory network deficits and hyperactivity.^209^ This points towards *APOE4*-mediated loss of inhibition as one driver of hippocampal hyperactivity.

Together, these findings from animal studies provide evidence that altered neural excitability contributes to both age-related memory dysfunction and Alzheimer’s disease progression, with a vicious cycle of protein accumulation and hyperexcitability. These parallel findings of hippocampal hyperactivity in ageing and Alzheimer’s models might reflect a high vulnerability of specific circuits to different conditions, but ageing itself might also make these circuits susceptible for Alzheimer’s disease-related hyperactivity.

### Increased fMRI activation is associated with worse cognitive outcomes and clinical progression in humans

Several cross-sectional studies specifically assessed the relationship between hyperactivation and cognition in humans, and converging lines of evidence suggest an association between increased activity and worse cognition.^17,69,142,210-212^ Findings in cognitively normal OAs, for example, suggest a link between deficits in inhibitory tone of the anterior hippocampus and attenuated performance improvement on a spatial learning task.^69^ A study by Maass *et al*.^17^ showed that tau-related fMRI hyperactivation during object discrimination is associated with a loss of domain-specific activation in posterior-midline regions that is in turn linked to poorer mnemonic discrimination in cognitively unimpaired OAs. In the same cohort, resting-state connectivity also revealed decreased segregation of anterior-temporal (object) and posterior-medial (scene) networks, which are associated with more tau and Aβ, respectively.^170^ This suggests that Alzheimer’s pathology contributes to neural dedifferentiation of domain-specific networks in ageing, which in turn may contribute to age-related cognitive decline.^213^ In patients with MCI, increased fMRI activation in dentate gyrus/CA3 was observed comitant to reduced dentate gyrus/CA3 and CA1 volume^142^ and worse mnemonic discrimination performance.^142^ Moreover, hippocampal hyperconnectivity has been associated with worse associative memory performance in patients with MCI^28^ as well as worse mnemonic discrimination performance within cognitively normal OAs.^119^

A few studies have investigated how increased fMRI activation and hyperconnectivity predict cognitive changes longitudinally. In an early fMRI study that investigated clinical progression of patients with MCI, there was a greater spatial extent of novelty-related activity in parahippocampal gyrus in patients who progressed to dementia over 2.5 years.^18^ Furthermore, baseline and longitudinally sustained hyperactivation was associated with cognitive decline over a 3-year period in Aβ-positive compared with patients with Aβ-negative MCI, independent of hippocampal volume.^165^ These findings suggest that fMRI hyperactivity is associated with increased risk for clinical progression. Another possibility, however, pertains to hyperactivity as a potentially compensatory process, with greater activation and recruitment of distal brain areas acting as a mechanism to maintain brain function, as discussed next.

### Potential compensatory mechanisms related to fMRI hyperactivation

**Figure 4:**
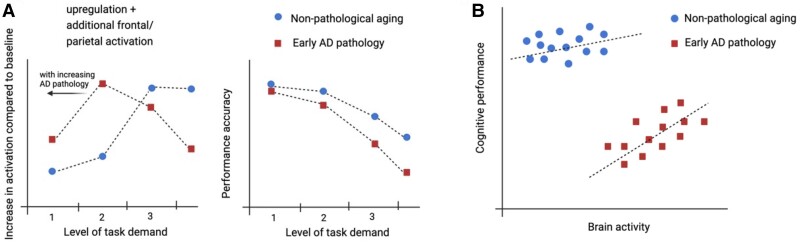
**Increased activity in the presence of Alzheimer’s disease pathology within the framework of compensation.** (**A**) Hypothetical demand–activity function in OAs with and without (early) Alzheimer’s disease pathology. In healthy older brains, activity increases with increasing task demands but finally declines due to limited neural resources. This non-linear demand–activity function is expected to be shifted to the left in the presence of early pathology due to earlier exhaustion of neural resources. Thus, at low or medium levels of task demand, increased compensatory activity would be observed in the presence of pathology during successful task performance. Figure adapted from Reuter-Lorenz and Cappell^215^ and Cabeza *et al*.^46^ Note that the inverted U-shape curve in **A** refers to activity changes with manipulated task demand within a subject. (**B)** When assessing brain activity across subjects, the group of subjects with (early) pathology might show higher activity than those without. If compensatory for disease, higher activity would be expected to positively correlate with performance in the group of individuals with pathology. Of note, a negative correlation between cognition and pathology might be observed when including all individuals. Figure adapted from Cabeza *et al*.^46^

Increased fMRI activation was initially interpreted as a compensatory mechanism reflecting plasticity to maintain cognitive function at an optimal level in response to early neurodegeneration.^16,47,48,74,96,99,144,214^ A set of criteria have been proposed to consider hyperactivation (or hyperconnectivity) as compensatory.^46^ These criteria state that hyperactivation must emerge when neuronal resources to accomplish a given mental operation are diminished (e.g. neurodegeneration or pathology) and that it must benefit cognition. In other words, if increased activation is found in individuals with increased Alzheimer’s pathology and is positively correlated with performance (see Fig. 4), this is consistent with compensation.^46^

Various theories of functional compensation have emerged based on studies of cognitively unimpaired OAs,^216-218^ mostly focusing on frontally mediated compensation^216-219^ This increased frontal activity in the presence of maintained performance was interpreted as enhanced deployment of neural resources in ageing to meet task demands. Supported by these findings, the ‘compensation-related utilization of neural circuits hypothesis’ (CRUNCH)^215,220^ proposes short-term upregulation of activity comitant to increased task demands as a potential mechanism of compensation. Breakdown of this mechanism leads to less activation and an incapacity to meet such demands.^215,220,221^ Other related models, such as the ‘scaffolding theory of ageing and cognition’ (STAC),^222,223^ the ‘posterior–anterior-shift in ageing’ (PASA) model,^224,225^ ‘early to late shift in ageing’ (ELSA) model^226^ and the hemispheric asymmetry reductions (HAROLD) model^227,228^ each propose age-related functional compensatory reorganization in response to task demands. Nevertheless, these activity changes could also reflect dedifferentiation.^46,213^

The aforementioned models mainly focused on the compensatory role of increased fMRI activation in normal ageing. In OAs with early Alzheimer’s disease, one might expect a shift of the demand–activity function (inverted U-shape) to the left due to an earlier compensatory activity increase or upregulation with lower task demands. Notably, increased activity should be associated with successful (maintained) task performance within subjects (Fig. 4A) or correlate positively with performance in the presence of pathology across subjects to be considered compensatory (Fig. 4B). One key study supporting compensation in older people with Aβ deposition assessed the detail level of memory in a subsequent memory paradigm.^32^ Aβ-positive OAs showed hyperactivation in task-positive regions, mainly parietal clusters, compared with Aβ-negative OAs. This was related to more detailed memory encoding in the Aβ-positive group only. Several other studies conducted in individuals at risk of developing Alzheimer’s disease dementia also provided empirical evidence supporting this proposition. For instance, increased fMRI activation in patients with MCI has been observed with comparable performance to cognitively normal controls on experimental memory tasks.^16,99,144^ Other studies have found a positive correlation between the degree of hyperactivation or hyperconnectivity of the temporal lobe and cognitive performance in MCI^99,140,229-232^ and SCD^27-30^. Rare interventional studies in MCI have shown that increased activation in non-specialized areas was associated with better memory performance following a cognitive intervention protocol.^233,234^

However, it is important to note that some studies have proposed a detrimental effect of fMRI hyperactivation on cognition. This is mainly based on the observation of negative correlations between hyperactivation and memory performance^14^ or the co-localization of hyperactivation and pathology.^17,34,66^ A set of experimental and randomized clinical trials have demonstrated that the levetiracetam-induced reduction of hippocampal hyperactivation improves memory performance in individuals with MCI,^19,49,235-237^ providing strong support for hyperactivation as a pathological biological state.

Several integrative models attempted to reconcile these findings.^6,8,117,238^ Jones *et al*.^6,8^ proposed a ‘cascading network failure model’ in which the focal deposition of tau pathology in the MTL triggers a local functional destabilization taking the form of hyperactivation or hyperconnectivity, followed by a global compensatory response from Aβ-processing areas forming the DMN. Aβ saturation would mark the breakdown of global compensation, allowing for tau to expand outside of the MTL, leading to a sequence of failure across cognitive networks. This model and others^47,117^ thus suggest that compensatory and pathological hyperactivation can co-exist in space and time and may vary depending on the disease stage. This notion has received empirical support from separate studies.^15,27,32,239,240^

## Proposed model of hyperactivation

Despite the contributions described above, an integrative model of task-based fMRI hyperactivation within the context of ageing and the pathological cascade of Alzheimer’s disease is lacking. Indeed, current models either rely on resting-state protocols and did not focus on the relationship between hyperactivation and cognition, lacked Alzheimer’s disease biomarkers and/or did not clearly delineate how the relationship between ageing, hyperactivation, cognition and Alzheimer’s disease pathology unfolds across the disease course.

**Figure 5:**
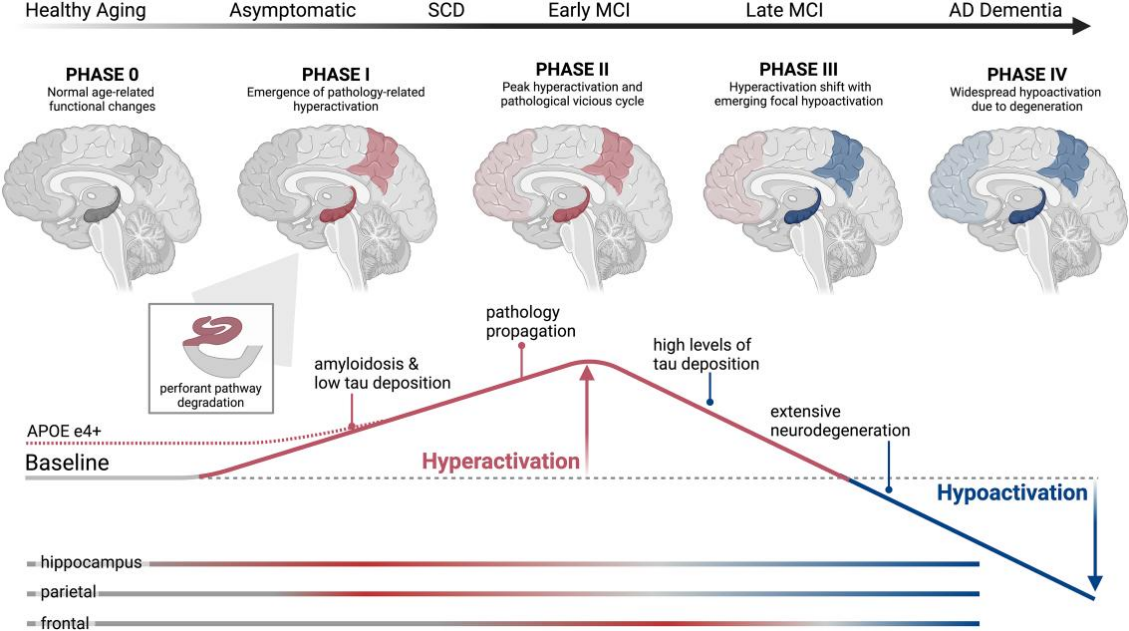
**Proposed model of hyper- and hypoactivation in the Alzheimer’s disease pathological cascade.** In Phase 0, healthy ageing is characterized by functional changes (*baseline*, grey) in comparison with younger adults, although these changes are not pathological in nature. In contrast, genetic predisposition to Alzheimer’s disease (i.e. *APOE4* genotype) may cause a prolonged state of increased activation across mid- to late life (red dotted line). In Phase I, age- and/or genetic-related functional changes predispose certain regions to pathology accumulation (i.e. hyperphosphorylated tau in MTL and Aβ in medial parietal lobe). This pathology accumulation coincides with the emergence of task-based hyperactivation (red), defined as increased activation contrasted against healthy OAs, which is evident when probed with episodic memory tasks. Hyperactivation first occurs in the hippocampus, particularly within dentate gyrus/CA3, due to tau-related perforant path degeneration (see inset box) and in parietal regions due to Aβ-related effects. Hippocampal hyperactivation might be probed with mnemonic discrimination tasks or repetition suppression, whereas a loss of suppression in posterior-midline (DMN) regions might be probed in various externally focused cognitive tasks. However, overt memory impairment is not yet evident at this stage. In Phase II, disconnection between the MTL and parietal lobe results in exaggerated hyperactivation, as well as accelerated expansion of pathology in a vicious cycle. This peak of hyperactivation is associated with SCD and early MCI. In Phase III, a tipping point of high levels of tau pathology ultimately leads to neuronal silencing and neurodegeneration, resulting in hypoactivation (blue) which first emerges in the hippocampus and parietal lobe. Simultaneously, a shift in hyperactivation to other regions (e.g. frontal lobe) occurs that might be observed for instance in working memory or cognitive control tasks. Finally, in Phase IV, widespread pathology and neurodegeneration leads to further hypoactivation that encompasses large-scale cortical regions and networks, resulting in overt cognitive impairment characteristic of Alzheimer’s disease dementia.

Here, we propose a model of task-based fMRI activity changes over the course of ageing and the Alzheimer’s disease pathological cascade that serves as a testable operational framework (Fig. 5). We describe a transition from normal ageing (Phase 0) to hyperactivity coincident with pathology accumulation in the early stages of Alzheimer’s disease (Phases I and II), which ultimately yield to hypoactivation, neurodegeneration and cognitive impairment (Phases III and IV). Our model builds upon foundational models of hyperactivation and large-scale network failures (e.g. see^6,117^) and models of activation changes in ageing.^215,222,223^ Critically, it bridges key insights and evidence spanning from mechanistic animal research to new human task-based fMRI studies tying hyperactivation patterns to biomarkers of Alzheimer’s pathology reviewed above. This model provides a hypothesized conceptual framework that has not yet been fully tested, prompting future research to design additional human fMRI studies to better elucidate the role of hyperactivation in the pathogenesis of Alzheimer’s disease.

The first element to consider pertains to the natural, age-related changes in functional brain systems occurring over the course of ageing. This corresponds to Phase 0 of our model, when observed changes are consistent with a normal ageing trajectory and not associated with any pathological change. However, these naturally occurring changes, which appear to occur within the MTL and parietal regions, decrease the resistance of these brain networks to late-life neurodegenerative diseases and in particular Alzheimer’s disease. The reasons why a specific network first succumbs to pathology are unclear, although developmental factors have been suggested as predisposal factors across Alzheimer’s disease variants.^122-124^ In the context of the canonical, amnestic variant of Alzheimer’s disease, the *APOE4* allele is known to render the temporal lobe vulnerable to pathology and neurodegeneration.^241^ The presence of this allele might interact with age-related processes and confers a higher vulnerability of memory systems to Alzheimer’s disease pathology. This mechanistic pathway seems to be at least partially mediated by life-long higher levels of activation as seen in young *APOE4* carriers^25,26,153-155,242,243^ and *APOE4*-related inhibitory network dysfunction in animal models.^209^

Phase I, which reflects the transition from normal to pathological ageing, is characterized by an increase of activation that is closely linked to early and abnormal accumulation of Aβ and tau pathologies. There are no noticeable clinical symptoms at this point; however, this early hyperactivation may be tied to worse performance on sensitive and specific tasks designed to probe the function of these hyperactive regions. The early accumulation of tau pathology within the entorhinal cortex is associated with a functional disruption of the pathways providing input to the hippocampus, leading to local functional isolation and hyperactivation of the hippocampus.^171^ This hippocampal hyperactivation is most evident when using task-related paradigms tapping into processes involving hippocampal circuits such as mnemonic discrimination,^34^ repetition suppression^36^ and associative memory. In concordance with the cascading network failure model,^6,8^ this local dyshomeostasis triggers a global and transient compensatory response in Aβ-processing areas forming the DMN.

Phase II corresponds to the transition from a purely asymptomatic stage to the onset of SCD and early MCI. From a biological perspective, hyperactivation reaches its peak and is widely distributed across frontal, parietal and temporal areas of the brain. The self-perpetuating, vicious cycle between Aβ, tau and functional hyperactivation is highly active. Task-related hyperactivation is observed outside of the hippocampus and can be elicited using tasks evoking cognitive processes that are not only hippocampal-dependent but also engage related networks (e.g. item memory, working memory, cognitive control and attention). Hyperactivation in areas known to exhibit early accumulation of pathology such as the hippocampus and precuneus likely reflect biologically detrimental processes, while hyperactivation in non-specialized areas and/or regions without pathology, such as the frontal lobe, may represent compensatory mechanisms.^28,32^ A careful examination of the relationship between the levels of activation in these areas and cognitive performance helps in determining the nature of this relationship.^48^

Phase III marks a breakdown of compensatory mechanisms and a saturation of tau pathology in the MTL, with tau deposition also found in neocortical regions. Clinically, this phase corresponds to late MCI or mild dementia. A tipping point is reached where tau pathology induces neuronal silencing and neurodegeneration, which relates to hypoactivation in the areas initially showing hyperactivation such as the hippocampal formation.^244^ As regions transition from hyperactivation to hypoactivation, they pass through a level of ‘pseudo-baseline’ activation, which may result in temporarily similar levels of activation compared with normal ageing adults (Phase 0). Residual and likely unsuccessful compensation mechanisms may still be at play,^46^ resulting in hyperactivation in remote areas not traditionally associated with the cognitive process being assessed.

Finally, Phase IV is associated with widespread hypoactivation, which may now also encompass regions such as the frontal lobe, which stems from widespread tau-related neurodegeneration and a decrease in functional network strength. Clinically, this phase corresponds to overt clinical symptoms associated with dementia.

Additional important points must be mentioned about the proposed model. First, it is unknown whether abnormal functional increases or MTL tau accumulation is the initiating event of the cascade, and there may be different trajectories and order of events between individuals. Longitudinal studies spanning the neuroimaging and molecular biology realms are required to answer this question. Further, it is possible that hyperactivation may first occur outside of the MTL, which may be more sensitively detected with task-based paradigms taxing cognitive processes other than memory. Although *APOE4* genotype is cited as a predisposing factor to pathological hyperactivation, the causes surrounding the emergence of this biological phenomenon are largely unknown. It is also important to keep in mind that this framework mostly relies on cross-sectional and methodologically limited studies. This model is primarily conceptual and calls for future work in ageing and Alzheimer’s disease to specifically test hypotheses generated from this model to further refine our understanding of large-scale systems supporting memory processes.

Another aspect that deserves mention is how this proposed framework applies to non-memory systems selectively degenerated in atypical variants of Alzheimer’s disease. While studies on this topic are more scarce and have mostly relied on resting-state fMRI, they revealed that networks become disrupted in a phenotype-specific manner. For instance, studies have found that the visual, language and control networks are functionally compromised in posterior cortical atrophy,^245-248^ logopenic aphasia^247-249^ and dysexecutive Alzheimer’s disease,^8^ respectively, while the DMN^8,239,250,251^ is commonly disrupted across these variants. These findings support the notion of shared large-scale pathophysiology across all Alzheimer’s disease variants and suggest that our proposed framework should apply to non-memory systems targeted in atypical Alzheimer’s disease phenotypes. However, answering this question requires design of studies specifically addressing the question of hyperactivation using task-based paradigms in these less commonly encountered clinical presentations.

Given the open questions and ample research still needed to further substantiate this hypothesized model, we encourage the design of innovative new task-based fMRI studies across the continuum of ageing and Alzheimer’s disease to provide additional evidence supporting or conflicting with the biological mechanisms outlined in this framework. We review methodological challenges and compelling future directions for the study of task-based fMRI hyperactivation in the field of Alzheimer’s disease in the following section.

## Challenges and future directions

### Limitations and recommendations

Task-based fMRI provides a widely available, non-invasive tool to measure local functional brain changes in response to a specific cognitive task. However, several methodological limitations have to be considered (summarized in Boxes 1 and 2). While the fMRI activation reflects changes in deoxyhaemoglobin concentrations in response to neural activity, the BOLD signal can also be affected by non-neuronal vascular changes. However, the contribution of vascular changes, which are common in ageing and disease, to hyperactivity remains largely unknown (see Box 2). Future studies should characterize and distinguish the contribution of vascular and neuronal influences to fMRI hyperactivation in multimodal designs by incorporating measures of cerebrovascular function (e.g. blood flow or cerebrovascular reactivity) and measures of activity (magneto- or electroencephalography) in the same subjects.^261^

Box 1 **Methodological challenges of fMRI-based hyperactivation**

- The fMRI BOLD signal reflects changes in deoxyhaemoglobin driven by localized changes in brain blood flow and blood oxygenation, which are coupled to underlying neuronal activity via neurovascular coupling. Thus, BOLD fMRI is an indirect measure of neuronal activity.
- MRI suffers from a variety of artefacts that can limit interpretation such as head motion,^252^ distortions and signal drop out particularly in the temporal and frontal lobes.
- The spatial resolution of 3 T fMRI is limited to a 1.5 mm isotropic resolution, which does not allow for differentiating between CA3 and DG or between input and output layers in the hippocampal–entorhinal circuitry.
- fMRI exhibits a low temporal resolution, resulting from a mismatch in the slower onset of the BOLD response and underlying haemodynamic response, which restricts measurement of temporal brain activity.^253^
- Task-based fMRI assesses relative BOLD responses between task conditions by subtraction, and there is no inherent baseline in traditional fMRI studies.^254^ ‘Activation’ or ‘deactivation’, therefore, always refers to the specific contrast.
- The use of different paradigms, different stimuli, contrasts and baselines limits the comparability between studies and might be one factor for inconsistencies across studies (e.g. only a few studies including biomarkers were performed during retrieval^45^).
- There is high variability in processing and analysis of fMRI data across studies and an urgent need for harmonization of analysis pipelines for better comparability.^255^

Box 2 **Potential non-neuronal factors contributing to hyperactivation**

Several additional factors have also been observed to contribute to altered fMRI activation in both ageing and Alzheimer’s disease and thus should be considered as pertinent in this context. While not exhaustive, these and potentially other contributing factors need to be accounted for in future research aimed at understanding hyperexcitability across the Alzheimer’s disease spectrum:

- Increased task-dependent BOLD signal in cognitively normal OAs versus young adults has been observed independent of changes in glucose metabolism, suggesting a non-neural origin to increased BOLD signal.^256^
- Microglia or astroglia activity might contribute to BOLD signal changes independent of neuronal activity (e.g. due to oxygen consumption^257^). The contribution of astroglia and microglia activity to fMRI-based hyperactivity in humans remains largely unknown.
- Though ageing and Alzheimer’s disease are associated with atrophy in regions typically showing fMRI-based hyper- and hypoactivity, structural differences in volume are usually not accounted for.^258^
- Modulatory effects on fMRI activation intensity have been reported to be related to cortical curvature and depth and macro-vasculature^259^ with vascular and venous architecture both affecting fMRI activation variability in humans.^259,260^
- Microvascular alterations can affect small vessel integrity, cerebral blood flow or reactivity/pulsatility—all common in old age and increased in Alzheimer’s disease, which could alter the BOLD signal without changes in underlying neural activity^261,262^
- Cardiovascular pulsations are thought to be a modulator of elevated BOLD signal in Alzheimer’s disease,^263^ thereby modulating fMRI responsivity independently of underlying neuronal contributions to the BOLD signal.

Similarly, the contribution of microglia or astrocytic activity to increased fMRI activation in humans is largely unknown (Box 2). Recent PET-fMRI studies in patients with dementia reported associations between microglia activation and altered connectivity^264^ as well as increased task-based fMRI activation independently of Aβ burden.^265^ Furthermore, studies combining glia-PET and ^18^F-fluorodeoxyglucose (FDG)-PET suggest that astrocytes and microglia contribute to changes in glucose metabolism^266,267^ in Alzheimer’s disease. Future studies combining fMRI with PET measures of neuroinflammation (e.g. using TSPO-PET tracers) and concurrent measures of glucose metabolism^256^ could help to elucidate the question about how glial activation affects the BOLD signal and how it might contribute to measures of hyperactivation. In addition, hypermetabolism on FDG-PET has been documented by a handful of studies in the early stages of neurodegenerative diseases,^113-116^ although this could reflect other biological parameters than hyperactivity such as microglia activation as noted above. Nonetheless, this modality has the potential to provide additional information about hyperactivation and could be used to track disease and/or therapeutic effects on functional network physiology.^268,269^

The cross-sectional nature of the vast majority of fMRI studies hinders our capacity to examine causal links between hyperactivation and the pathobiological and cognitive evolution of Alzheimer’s disease. Additional longitudinal multimodal neuroimaging studies are needed to examine the temporal and spatial emergence and progression of hyperactivity in relationship to regional Aβ and tau to improve our capacity to situate hyperactivation along the pathological cascade of Alzheimer’s disease and to substantiate our proposed model. Additionally, parallel assessment of task-based fMRI activation and functional connectivity changes is critical to assess whether focal hyperactivation antedates large-scale functional disruption or is concomitant to it.^28,270^

Notably, task-based fMRI is an inherently contrastive methodology (Box 1) where univariate task activity is usually measured as comparison between the condition of interest (e.g. novel stimuli) relative to a baseline (e.g. fixation or familiar stimuli). However, the use of different paradigms/stimulus material, different contrasts, as well as different baselines^254^ limits the comparability of studies and might be one factor for inconsistencies across studies.^45^ For instance, increased hippocampal activity in OAs with high relative to low tau burden has been observed in a mnemonic discrimination task with novel and repeated images when collapsing across all conditions relative to a perceptual baseline.^17^ A follow-up analysis^36^ revealed that the increased tau-related activity in the hippocampus was most prominent for the repeated stimuli. This suggests that previous findings of reduced hippocampal novelty activity (novel < familiar) could also be driven by increased fMRI activation for familiar information.^151,271^ With respect to the posterior-midline regions, increased activity has been broadly reported when comparing different tasks versus rest/fixation conditions (see Box 1), where increased fMRI activation often reflected a loss of suppression (e.g. during encoding^21,31,78^) or a loss of modulation/habituation with repetition.^78^ However, task-related hyperactivation might not be seen at very high task demands.^40^ Future studies should consider the influence of task demands^46,164^ and include adequate baselines, as it has been demonstrated that even relatively short periods of rest or fixation engages the DMN and cannot be considered as a baseline of null activity.^254^

While animal studies point towards specific hyperactivity in the CA3 auto-associative network and altered input from superficial entorhinal layers via the perforant pathway in mouse models of Alzheimer’s disease, fMRI studies in humans are limited by the spatial resolution of fMRI. Most previous fMRI work on MTL hyperactivity in ageing and Alzheimer’s disease has been conducted with field strengths of 1.5 or 3 Tesla, which did not allow to separate activity between CA3 and DG or different layers in the hippocampal–entorhinal circuitry (see Box 1). With the increasing availability of ultra-high-field 7 Tesla and even 9.4 Tesla scanners, combined with novel advances in neuroimaging sequences such as vascular space occupancy,^272^ acceleration techniques such as multiband imaging^273^ and improved motion correction and post-processing,^274^ future studies will be able to measure activity and connectivity at a submillimetre resolution^275^ in OAs and patients. Laminar and subfield imaging in OAs or patients characterized by their Aβ and tau biomarker profile will allow us to test circuit specific hypotheses of Alzheimer’s disease-related hyperactivity in the MTL.

Furthermore, translational studies that assess Alzheimer’s disease-related hyperactivity in parallel in human and animal models are needed. This could be done, for instance, by combining direct measures of neuronal activity (e.g. electrophysiology or calcium imaging) with BOLD fMRI in rodent models^276,277^ or primates, and correlating findings with human fMRI data. Finally, combining MR spectroscopy for regional estimation of GABA and glutamate with fMRI^278,279^ could give further insight into the synaptic contributions to fMRI hyperactivity. Overall, these studies could bridge across scales and advance our understanding of the underlying basis of increased BOLD signal.

### Hyperactivity as therapeutic target

One promising area lies in characterizing the translational implications of hyperactivation and functional network disruption, particularly for the development of novel therapies aimed at modulating large-scale functional physiology. Past and ongoing studies suggested that the use of levetiracetam is associated with reduction of hyperactivity in hippocampal and parietal areas^187^ and beneficial effects on memory performance in patients with MCI.^49,235,236^ Recently, the results from the HOPE4MCI trial were published.^280^ This was a Phase 2b trial targeting the reduction of hippocampal hyperactivity and improvement of memory in patients with MCI with a low dose of levetiracetam. While there was no significant difference after 18 months in global cognition, stratified analyses by APOE4 status indicated a beneficial yet non-significant effect in non-carriers. It is of note that the conclusions from this study are limited by the small sample size. The use of selective GABA-Aα5-positive allosteric modulators is also a promising therapeutic approach for reduction of hyperactivity, but these efforts are currently in preclinical development.^199^ Future randomized controlled clinical trials targeting hyperactivity should consider stratification by pathology burden and *APOE4* genotype and also include functional outcome measures of cerebral hyperactivation (such as fMRI or EEG) to validate the mechanistic effect of the drugs.

Complementary to these pharmacological interventions, non-invasive brain stimulation interventions directly targeting brain networks could prove useful in reducing hyperactivity and slowing cognitive decline.^281^ Recent work suggests that non-invasive transcranial magnetic stimulation may improve cognition in patients with Alzheimer’s disease.^282^ In a recent randomized, sham-controlled trial in patients with Alzheimer’s disease dementia, 24 weeks of precuneus transcranial magnetic stimulation was associated with attenuated cognitive decline and stable local cortical excitability as measured by EEG.^283^ Another promising brain stimulation technique is low-intensity transcranial focused ultrasound which can penetrate the skull and dura and modulate neural activity (via mechanical action on cell membranes) also in deep brain structures, such as the hippocampus or entorhinal cortex.^284,285^ A recent study in young adults applied transcranial focused ultrasound to the MTL, which was found to selectively modulate perfusion, fMRI activation and functional connectivity in the targeted entorhinal cortex and its network.^286^ Finally, a recent trial in patients with MCI has been initiated to test whether real-time fMRI neurofeedback is able to reduce hippocampal hyperactivity and thereby improve memory performance in patients with MCI.^287^ Despite a need for further in-depth research, hyperactivity seems to be a promising therapeutic target for Alzheimer’s disease and could potentially be paired with currently available disease-modifying treatments.^288,289^

## Conclusion

This review proposes that task-based fMRI hyperactivation is a fundamental feature of the Alzheimer’s disease pathological cascade. Hyperactivation may reflect large-scale and progressive dyshomeostasis of cognitive systems that may serve as an endophenotype between molecular pathology and clinical manifestations. While the causes of hyperactivation are yet to be fully understood, developmental factors and life-long effects of ageing may render memory systems particularly vulnerable to late-arising neuropathology. Our proposed model of hyperactivation describes a temporal sequence of functional abnormalities across the clinico-pathological spectrum of Alzheimer’s disease. This framework provides a foundation to formulate and test hypotheses aimed at a better understanding of Alzheimer’s disease from a complex system standpoint. Hopefully, a better understanding of the multi-scale interactions between misfolded proteins and large-scale systems translates into sorely needed interventions aimed at or incorporating hyperactivation and system-level physiology.

## Supplementary Material

fcae376_Supplementary_Data

## Data Availability

Data sharing is not applicable to this article as no new data were created or analysed in this study.
