# Supplementary material for: Cerebral hyperactivation across the Alzheimer’s disease pathological cascade
Source: Brain Commun. 2024 Oct 25;6(6):fcae376. doi: 10.1093/braincomms/fcae376 (PMC11542485; doi:10.1093/braincomms/fcae376)
Supplement: fcae376_Supplementary_Data [file fcae376_supplementary_data.pdf]

Supplementary Table 1. Overview of human task-based fMRI studies

Study	Sample	Biomarker	MRI methodology	Results
Bookheimer et al., 2000[1]	-16 OA *APOE4* (14 E3/E4, 2 E4/E4) (64y)<br>-14 OA *APOE* E3/E3 (63y)<br>-Longitudinal	-	-*Design*: alternation of 2 conditions: learning (6 x 7 trials), rest (6 x 30 sec), afterwards 6 blocks of recall<br>-*Task*: auditory paired-associates learning task, retrieval: first word is heard, second word silently recalled, training before<br>-*Contrast*: encoding v rest, recall v rest, learning and recall v rest	- No differences in word-pair task performance<br>-Task-related activation in left HC and PHC, PFC, ACC, left inferior frontal, left posterior temporal and parietal region: E4 > E3<br>-Potential compensation in *APOE4*<br>-Group-differences driven by differences in task-activation (learning and recall), not rest<br>-After 2y: positive correlation of baseline activation and verbal recall in memory selective reminding test (14 subjects with 8 *APOE4*)
Lustig et al., 2003[2]	-32 YA (22y)<br>-27 OA (76y)<br>-23 early AD (77y)	-	-*Design*: alternating blocks of 2 conditions: active semantic classification task (2x12 trials) and visual fixation (2x)<br>-*Task*: semantic classification judgment (living/ non-living) on visually presented words (incidental encoding), recognition immediately (no MRI)<br>-*Contrast*: task v fixation (frontal/ parietal ROIs)	-Task-related deactivation in lateral parietal regions: equivalent across groups<br>-Deactivation in medial frontal regions: YA > OA/ AD (higher activity in OA during task)<br>-Deactivation medial parietal (precuneus/ PCC): YA > OA >AD (higher activity in OA and AD during task)<br>-Temporal profile of medial parietal/PCC response suggested initial activation by all three groups<br>-YA: quickly reversed sign<br>-AD: maintained activation throughout the task block
Machulda et al., 2003[3]	-11 OA (79y)<br>-9 MCI (76y)<br>-9 AD (80y)	-	- *Design*: alternating blocks of 2 conditions: encoding task targets (4x3 trials) and foils (4x3 trials)<br>-*Task*: visual scene encoding: memorizing photographs (intentional encoding) training before, recall immediately, recognition after 5 min (no MRI)<br>-*Contrast*: -<br>(A series of one-sided rank sum pairwise comparisons were performed to test for differences in extent of task-activation between pairs of subject groups)	-Task-related MTL activation OA > MCI/ AD<br>-Recall performance correlated with extent of activation across all subjects, recognition did not
Sperling et al., 2003[4]	-10 YA (25y)<br>-10 OA (74y)<br>-7 early AD/ MCI (81y)	-	-*Design*: blocks of 3 conditions: novel face–name pairs (12x7), repeated face–name pairs (2 alternating pairs, each seen 49 times), and visual fixation<br>-*Task*: face–name association encoding: viewing faces on photographs with common names for 5 sec, (intentional encoding), face recognition and recall of corresponding name immediately (no MRI)<br>-*Contrast*: novel v fixation (NvF), repeated v fixation (RvF), novel v repeated (NvR)	-Task-related NvR activation in superior and inferior PFC: YA > OA<br>-Task-related NvR activation in parietal regions: OA > YA<br>-Task-related NvR activation in right HC:<br>YA > OA > AD<br>-Task-related NvR activation in medial parietal and right PCC: AD > OA
Johnson et al., 2004[5]	-11 OA (75y)<br>-12 MCI (79y)	-	-*Design*: mixed design of 2 conditions in 3 runs: repeated viewing of faces	-Adaptation slope MTL: OA more negative than MCI (less repetition suppression in MCI)

			(4x7 trials) and visual fixation<br>-*Task*: novel repeating face encoding: 3x4 gray-scale photographs of faces, each repeated 7 times (intentional encoding)<br>-*Contrast*: -<br>adaptation slope (time x activation interaction) (MTL ROI)	-OA: larger response to initial two presentations followed by gradual decline<br>MCI: subtle gradual increase in activity<br>-VBM: no relation of activation and gray matter probability
Greicius et al., 2004[6]	-14 YA (21y)<br>-13 OA (75y)<br>-13 mild AD (77y)	-	-*Design*: mixed design of 2 conditions: single and paired stimuli, pseudorandomized (4x15 trials)<br>-*Task*: sensory-motor processing task: button-press when stimuli presented (single or paired flashing checkerboard) (low cognitive demand)<br>-*Contrast*: -<br>(ICA DMN)	- Coactivation EC - HC: YA/ OA > AD<br>- Activation PCC, inferior parietal region: OA > AD
Dickerson et al., 2004[7]	-32 MCI (75y)<br>-Longitudinal<br>-Cohort: Harvard Medical School	-	-*Design*: alternation of 3 conditions per run: fixation, novel scenes (12 trials), repeated scenes (3x4 trials, same order)<br>6 runs, each: fixation, novel (6 trials), fixation, repeated, fixation, novel (6 trials), fixation, repeated, fixation (intentional encoding), baseline and follow-up after 2.5y (no MRI)<br>-*Task*: scene visual encoding task: remembering complex colored photographs, recognition after 20 min (no MRI)<br>-*Contrast*: novel v fixation (NvF), novel v repeated (NvR)<br>(MTL ROI)	-Task-related NvF/ NvR activation in HC/ PHC: activation correlated with better memory performance (not with additional CVLT)<br>-Task-related NvR activation in right PHC: activation correlated with greater clinical impairment, even after accounting for atrophy<br>- NvR: decliner group in follow-up (n=14): greater extent of right PHC fMRI activation but no difference in task performance<br>-Potential compensation<br>-No effect of *APOE4*
Bondi et al., 2005[8]	-10 OA *APOE4* (8 E3/E4, 2 E4/E4)(76y)<br>-10 OA *APOE* E3/E3 (76y)	-	-*Design*: alternation of 3 conditions: novel pictures (8 x 6 trials), repeated picture (control condition), fixation (4 blocks)<br>-*Task*: novelty encoding task, stimuli: color photographs of indoor/ outdoor scenes, repeated stimulus: 1 photograph of autumn leaves, 2AFC recognition after 10 min for the 48 novel stimuli (no MRI)<br>-*Contrast*: novel v repeated (NvR)	-No differences in recognition performance<br>-Task-related NvR activation in superior frontal, superior temporal, cingulate, right superior temporal region in E4<br>-Task-related NvR activation in occipital, inferior frontal, right PCC, left HC, left PHC in E3<br>-Task-related NvR activation in right superior parietal, left middle frontal gyrus, medial frontal gyrus, fusiform gyrus: E4 > E3
Dickerson et al., 2005[9]	-10 OA (72y)<br>-9 mild MCI (74y)<br>-10 prodromal AD (78y)<br>-Cohort: Harvard Medical School	-	-*Design*: see Sperling et al., 2003<br>-*Task*: face–name association encoding (intentional encoding), immediate name-face recognition (2AFC)<br>-*Contrast*: novel v repeated (NvR) (MTL ROI)	-Task-related NvR activation in HC: MCI > OA (no difference in EC)<br>-Task-related NvR activation in HC and EC: OA/ MCI > AD<br>-Atrophy in HC and EH: AD > OA/ MCI<br>-Performance recognition task: OA/ MCI > AD<br>-Across all subjects, greater EC activation in subgroup of 13 carriers of *APOE4* than in the 16 non-carriers<br>-Potential compensation

				- Across all subjects, task performance was best predicted by activation of right EC and HC and right HC volume<br>-Inverted U: increased MTL activation early (MCI), then decrease in activation with AD
Celone et al., 2006[10]	-15 OA (76y)<br>-15 mild MCI (75y)<br>-12 severe MCI (80y)<br>-10 AD (78y)<br>-Cohort: Harvard Medical School	-	-*Design*: see Sperling et al., 2003<br>-*Task*: face-name association encoding (intentional encoding), rating of 'fit' of name to face, face recognition and immediate name-face recognition (no MRI)<br><br>-*Contrast*: -<br>(ICA: activity correlated with task, Pos-TrC and Neg-TrC (TrC: task-related component))	-Pos-TrC: task-activations: HC, inferior PFC, fusiform, visual regions: all groups (activity HC: mild MCI > OA > severe MCI/ AD)<br>-Neg-TrC: task-deactivations: precuneus, PCC, lateral parietal and temporal cortex, ACC, MFC: all groups (deactivation precuneus/ PCC: mild MCI > OA > severe MCI/ AD)<br>-Across all subjects, positive correlation of extent of HC activation and parietal deactivation (and correlation with memory performance)<br>-Nonlinear trajectory of fMRI activation across the continuum of impairment: Inverted U-shape in MTL/ task positive region, but increased (de-)activation in mild MCI<br>-Potential compensation in MCI
Kircher et al., 2007[11]	-29 OA (68y)<br>-21 MCI (70y)	-	-*Design*: blocks of 2 conditions: encoding (2x90 trials), recognition (4x90 trials, 45 targets and 45 foils), after block of encoding followed two blocks of recognition, fixation between stimuli<br>-*Task*: encoding of words (intentional encoding), recognition directly after encoding block (MRI)<br>-*Contrast*: subsequent hits (sub. hits) v fixation, sub. hits v misses	-Sub. hits v fixation activation in HC/ MTL: MCI > OA<br>-No difference in amount of hits between groups<br>-Potential compensation in MCI<br>-Subsequent memory effect (sub. hits v misses): MCI > OA<br>-Activation MCI: fusiform, PFC, precuneus, PCC, occipital, middle temporal region
Wright et al., 2007[12]	-12 YA (24y)<br>-12 OA (71y)<br>-12 mild AD (72y)	-	-*Design*: 2 runs of Ekman faces: familiarization of 2 neutral faces, test phase of familiar and 8 novel fearful faces, fixation between stimuli<br>-*Task*: viewing of neutral and fearful faces, recognition memory test<br>-*Contrast*: familiar neutral v fixation, novel fearful v fixation	-Task performance: YA = OA = AD<br>-Task-related activation in amygdala novel fearful v fixation > familiar neutral v fixation in all groups<br>-Task-related activation in amygdala for both contrasts: AD > OA<br>-Correlation of responses to familiar neutral faces and neuropsychiatric symptoms in AD
Hämäläinen et al., 2007[13]	-21 OA (71y)<br>-14 MCI (72y)<br>-15 mild AD (73y)	-	-*Design*: alternation of 3 conditions in 6 runs: encoding (5 trials), cued recall of previous trials, visual fixation<br>-*Task*: associative encoding of novel picture-word pairs: name black-white line drawing with context-related cue word and recall it with cue, indicate with button press (intentional encoding), training before<br>-*Contrast*: encoding v fixation	-Task-related activation in posterior HC, PHC and fusiform gyrus: MCI > OA (no differences MCI v AD or OA v AD)<br>-Task performance: OA > MCI > AD<br>-VBM GMV HC: atrophy in anterior left HC: MCI > OA<br>atrophy whole HC: AD > OA<br>-Negative correlation of HC volume and PHC activation in MCI, not in OA/ AD
Miller et al., 2008 (PNAS)[14]	- YA (24y)<br>-OA (75y)	-	-*Design*: see Sperling et al., 2003<br>-*Task*: face–name association encoding (intentional encoding), rating of 'fit' of name to face, immediate name-face	-Subsequent memory effect (sub. hits v misses):<br>activations in HC: YA and OA similar in extent (and magnitude)<br>deactivations in precuneus: YA > OA (YA more deactivation in high conf. hits)

			recognition, confidence rating (high/ low) (no MRI)<br>-*Contrast*: subsequent high confidence hits v misses, trial type v fixation (HC, precuneus ROI)	-OA as a group did not demonstrate a differential pattern of deactivation between trial types (whole-brain analysis)
Miller et al., 2008 (J Neurol Neurosurg Psychiatry)[15]	-25 MCI (73y)<br>-Longitudinal	-	-*Design*: see Dickerson et al., 2004, annual behavioural follow-up for min. 4y<br>-*Task*: visual encoding task with scenes<br>-*Contrast*: novel v repeated (NvR) (HC ROI)	-Greater magnitude of HC activation predicted greater degree and rate of subsequent cognitive decline<br>-HC only brain region where activation predicted cognitive decline (whole-brain analysis)<br>-*APOE4* contributed significantly to prediction model
Pihlajamäki et al., 2008[16]	-29 OA (74y)<br>-15 mild AD (78y)<br>-Cohort: Harvard Medical School	-	-*Design*: see Sperling et al., 2003, trials divided into 6 instead of 7 runs<br>-*Task*: face–name association encoding (intentional encoding), rating of 'fit' of name to face, immediate name-face recognition (no MRI)<br>-*Contrast*: repeated v fixation (RvF) (MTL, parietal (precuneus, RSC, PCC) ROI)	-Task-related RvF activation in MTL, PFC, superior parietal: AD > OA (failure repetition suppression in AD)<br>-Task-related RvF deactivation in precuneus, right PCC, left lateral parietal: OA > AD<br>-Magnitude of RvF activation in MTL ROI: AD > OA<br>-Magnitude of RvF deactivation in parietal ROI: OA > AD<br>-Increased MTL RvF activation in AD related to disrupted parietal deactivation and to poor recognition performance
Rodda et al., 2009[17]	-10 OA (68y)<br>-10 SCI (64y)	-	-*Design*: alternating blocks of 2 conditions: active (encode) (9x8 trials) and comparison (control) (9x4 trials, 'wait')<br>-*Task*: verbal episodic memory encoding task: memorize nouns of a semantic theme per block by reading out loud (intentional encoding), training before, recognition after 12 min (no MRI, divided attention task in between) with 17 targets, 16 novel foils, 14 lures<br>-*Contrast*: encode v control	-Task-related activation in left lateral PFC:<br>SCI > OA<br>-No differences in task performance between groups<br>-Left PFC activation strength correlated with memory task performance<br>-Potential compensation in SCI
Sperling,et al., 2009[18]	-18 YA (24y)<br>-35 OA (77y) (13 had CDR 0.5)<br>-Cohort: Harvard Medical School	-PiB DVR in precuneus/ PCC<br>-PiB+: DVR >1.6, n =13 OA	-*Design*: see Sperling et al., 2003<br>-*Task*: face-name association encoding (intentional encoding), rating of 'fit' of name to face, immediate name-face recognition, confidence rating (high/ low) (no MRI)<br>-*Contrast*: fixation v "high confidence hit" (HCH) (PPC (precuneus/ PCC) ROI)	-Task-related extent of deactivation in DMN (precuneus, PCC, mPFC):<br>YA > OA PiB- > OA PiB+<br>(OA PiB+ showed increase in activity; similar to AD subjects, see Celone et al., 2006; Pihlajamaki et al., 2008)<br>-Functionally defined ROI PPC: highest amyloid burden and most significant DMN deactivation in YA: higher PiB DVR correlated with higher magnitude of activation
Filippini et al., 2009 [19]	-18 YA *APOE4*+ (28y)<br>-18 YA *APOE4*- matched controls (29y)	-	-*Design*: blocks of 2 conditions: new stimuli (6x8 trials), previously learned stimuli (6x8 trials), mixed with fixation<br>-*Task*: picture encoding task<br>-*Contrast*: novel v previously learned	-No differences in performance<br>-Task-related activation in right HC: E4+ > E4- (also for novel v fixation)
Dennis et al., 2010[20]	-12 YA *APOE4*+ (22y)	-	-*Design*: 3 runs of each 10 blocks of 2 conditions:	-No differences in performance<br>-Task-related activation in HC, PHC: E4+ > E4-

	-12 YA *APOE*-matched controls (21y)		objects (5x8 trials), fixation (5 blocks)<br>-*Task*: encoding task (incidental encoding), stimuli: emotionally neutral pictures, recognition after 24h for 200 words: 120 previously seen pictures, confidence rating (no MRI)<br>-*Contrast*: subsequently remembered v forgotten (MTL ROI)	
Quiroz et al., 2010[21]	-20 YA PSEN1 mutation (34y)<br>-19 YA non-carrier matched controls (34y)<br>-Cohort: Familial Colombian Alzheimer's disease population	-	-*Design*: see Sperling et al., 2003, with hispanic faces and names<br>-*Task*: face-name associative encoding (intentional encoding)<br>-*Contrast*: novel v repeated (HC ROI)	-No differences in performance<br>-Task-related novel v repeated activation in right anterior HC: YA carrier > YA non-carrier (driven by higher activation for novelty in YA mutation)
O'Brien et al., 2010[22]	-21 OA CDR 0 (73y)<br>-30 OA CDR 0.5 (76y)<br>-Longitudinal<br>-Cohort: Harvard Medical School	-	-*Design*: see Sperling et al., 2003, baseline and follow-up after 2y<br>-*Task*: face-name association encoding (intentional encoding), immediate name-face recognition (no MRI), recognition: baseline 14 trials and follow-up all 84 trials so no investigation of longitudinal memory performance<br>-*Contrast*: novel v repeated (NvR) (HC ROI)	-Task-related longitudinal decrease in NvR extent of activity in right HC: CDR 0.5 (no changes in activity in CDR 0) (anterior right HC then used as functional ROI)<br>-Magnitude of activation in functional HC ROI at baseline: fast CDR decliners > stable CDR 0 group<br>-Loss of magnitude of activation in functional and anatomical HC ROI: CDR decliners > CDR non-decliners<br>-Decline in magnitude of activation is correlated with decline in CDR and greater magnitude of baseline activation<br>-No effect of *APOE4*
Clément & Belleville, 2010[23]	-14 OA (67y)<br>-26 MCI (68y) (higher-cognition and lower-cognition group, split-median on dementia rating MDRS)	-	-*Design*: blocks of 3 conditions: semantically related (8x9 trials), semantically unrelated word pairs (8x9 trials), visual fixation<br>2 runs, each included: 8 blocks of fixation, 8 blocks of encoding, 8 blocks of recognition (8 word-pairs: 4 targets, 4 foils)<br>-*Task*: verbal learning of word pairs (intentional encoding), training before<br>-*Contrast*: related v fixation, unrelated v fixation (HC ROI)	-Memory recognition performance: OA/ MCI high-cog. > MCI low-cog.<br><br>-Additional task-activation in the right dlPFC (both conditions) and in the left PHC/ HC (only unrelated condition): MCI high-cog.<br>-Potential compensation in MCI high-cog.<br>-No additional task-related prefrontal activation, decreased activation in posterior areas: MCI low-cog.<br>-Higher magnitude of left HC activation correlated with higher cognition score (MDRS)
Yassa et al., 2010[24]	-10 OA (75y)<br>-10 aMCI CDR 0.5 (76y)	-	-*Design*: pseudorandomized alternations of 3 conditions in 6 runs: novel foils (44 trials per run), repeated (16 trials per run), lures (similar but not identical) (16 trials per run)<br>-*Task*: explicit 3AFC pattern separation task: continuous recognition of color photos of objects, judgment: is stimuli new, old or similar to previous one<br>-*Contrast*: lures subsequently called similar	-Performance new/ repeated: no difference<br>-Performance pattern separation (lures as similar, not old): OA > aMCI<br>-Task-related sLS/ LS activation in left CA3/ DG: aMCI > OA<br>-Activation negatively correlated with overall separation performance<br>-General task-hyperactivity effect across HC subregions: aMCI > OA<br>-Task-related sLS activation in left EC: OA > aMCI

			(sLS) v lures subsequently called old (sLO) (timepoint: 1st presentation - encoding), lures called similar (LS) v lures called old (LO) (timepoint: 2nd presentation - retrieval) (MTL ROIs)	
Pihlajamaki et al., 2011[25]	-15 YA (23y)<br>-30 OA CDR 0 (74y)<br>-30 MCI CDR 0.5 (77y)<br>-15 mild AD CRD 1 (78y)<br>-Cohort: HABS	-	-*Design*: see Sperling et al., 2003<br>-*Task*: face–name association encoding (intentional encoding), rating of 'fit' of name to face, immediate name-face recognition<br>-*Contrast*: repeated v fixation (RvF), novel v fixation (NvF)	- RvF magnitude of activation in anterior MTL inversely correlated with performance in recognition performance across all subjects<br>-Task-related NvF-RvF magnitude of activation difference in anterior MTL: YA > OA > MCI > AD<br>-MTL RvF extent of activation negatively correlated with word-list delayed recall (RAVLT)
Putcha et al., 2011[26]	-18 OA CDR 0 (74y)<br>16 MCI CDR 0.5 (75y)<br>-Cohort: HABS	-	-*Design*: see Sperling et al., 2003<br>-*Task*: face–name association encoding (intentional encoding), rating of 'fit' of name to face, immediate name-face recognition, confidence rating (high/ low) (no MRI)<br>-*Contrast*: high confidence hit v fixation (HCHvF) (HC ROI)	-Task-related HCHvF magnitude of activation in HC:<br>MCI > OA (no difference in HC volume)<br>-within this MCI group, increased HCHvF activation in HC correlated with cortical thinning in AD-signature regions<br>-Within the OA group, increased HCHvF activation in HC was negatively correlated with cortical thinning in a subset of regions, including the superior parietal lobule
Erk et al., 2011 [27]	-20 OA (67y)<br>-19 SCI (68y)	-	-*Design*: encoding: blocks of 2 conditions (2x): face-profession pairs (4x4 trials), control head contours (4x6 trials)<br>recall: blocks of two conditions (1x): apprentice or student during training for job (4x4), control (4x6)<br>recognition: blocks of two conditions (1x): 2AFC of profession (4x4), control (4x4)<br>-*Task*: associative face-profession encoding (incidental encoding), targets: imagine person in this profession, rating of 'fit'; controls: size judgment of ears), recall, recognition,<br>additional working memory task: n-back task (all MRI)<br>-*Contrast*: task v control, 2-back v 0-back (HC, dlPFC ROI)	-Performance in tasks: no difference<br>-Task-related encoding, recognition, working memory activation: no difference (whole-brain analysis)<br>-Task-related recall v control activation in right HC: OA > SCI<br>-Task-related recall v control activation in dlPFC: SCI > OA<br>-Positive correlation magnitude of activation right dlPFC and subsequent recognition performance in SCI<br>-Potential compensation in SCI
Rodda et al., 2011[28]	-10 OA (68y)<br>-11 SCI (65y)	-	-*Design*: alternating blocks of 2 conditions: divided attention (5x16 trials), control attention (5x)<br>-*Task*: divided attention task: pairs of stimuli: visual (letters) and auditory (spoken numbers), targets: q and 8, control: visual and auditory numbers (target: 8)<br>-*Contrast*: divided attention v control attention (dAvcA)	-Performance in task: no difference<br>-Task-related dAvCA activation in left MTL, bilateral thalamus, PCC and caudate:<br>SCI > OA<br>-Potential compensation in SCI
Yassa et al., 2011[29]	-17 YA (23y)<br>-10 OA (75y)	-	-*Design*: see Yassa et al., 2010<br>-*Task*: pattern separation task, additional mnemonic	-Performance regarding false alarms (lures called old): YA > OA (otherwise no differences in performance)

			similarity task of pictures (each 64 new, lures, old) (no MRI)<br>-*Contrast*: lures called similar v lures called old; for both subsequent memory (first presentation) and lure presentation (second presentation) (MTL ROIs)	-Task-related magnitude of activation in DG/ CA3: OA > YA (both contrasts) (=increase in difference between correct rejections and false alarms)<br>-DG/ CA3 activation is negatively correlated with separation performance
Bakker et al., 2012[30]	-17 OA CDR 0 (69y)<br>-17 aMCI CDR 0.5 (73y)	-	-*Design*: see Yassa et al., 2010<br>double-blind randomized clinical intervention: placebo and 125 mg levetiracetam aMCI group (switch for second time of fMRI task)<br>-*Task*: explicit 3AFC pattern separation task<br>-*Contrast*: lures called similar v novel foils (= correct pattern separation v implicit baseline) (MTL ROIs)	-Performance regarding false alarms (lures called old):OA > aMCI<br>-Task-related activation in DG/ CA3: aMCI > OA<br><br>-With levetiracetam:<br>-HC activation: aMCI = OA<br>-EC activation: OA > aMCI<br>-Task performance improved (more lures called similar instead of old)
Mormino et al., 2012[31]	-15 YA (23y)<br>-45 OA (PiB-: 75y, PiB+: 76y)<br>-Cohort: BACS	- PiB DVR<br>-PiB+: DVR > 1.08, n = 15 OA	-*Design*: alternation of 2 conditions in 4 runs: viewing of pictures (4x50 trails) and fixation (4x50 trials)<br>-*Task*: episodic memory-encoding of natural scenes (incidental encoding) with judgment if water is in image,<br>recognition 15 min after task (no MRI) (200 targets, 100 foils, high/ low confidence recognition rating)<br>-*Contrast*: high-confidence hits v misses (DM effect: difference due to subsequent memory)	-Performance in recognition task: no difference<br>-Task-related DM activation in vlPFC, lateral occipital/ parietal, posterior/ inferior temporal, right PHC/ HC: YA > OA<br>(on average across the regions and specifically in right HC: PiB+ > PiB-)<br>-Task-related DM deactivation in mPFC, dlPFC, posteromedial, angular, lateral temporoparietal cortex:<br>YA > OA (YA more deactivation during hits, no difference in misses) (no difference regarding PiB)<br>-Positive correlation of deactivation and task memory performance in OA<br>-Positive correlation of activation in PiB+ and memory scores<br>-Potential compensation<br>- PiB+/-: no difference in *APOE*-status or recognition performance
Bejanin et al., 2012[32]	-12 OA (59y)<br>-11 AD (77y)	- H^2O^{15} PET	-*Design*: blocks of 5 conditions: rest (4x2 min), reading (2x24 trials), encoding (2x24 trials), stem-cued recall of targets (2x24 trials), stem-cued completion (2x24 trials)<br>-*Task*: encoding of verbal items (intentional encoding), reading and completion as baseline without encoding or recall, living/ non-living judgment in reading and encoding condition, cued recall with first two letters of word, completion of two letters with first word that comes to mind, answers spoken aloud<br>-*Contrast*: encoding v reading, recall v completion, target (encoding or recall) v rest	-Performance in recall task: OA > AD<br>-Task-related encoding v reading activation in PHC, HC, left ACC: OA > AD<br>-Task-related encoding v reading activation in MFG, inferior parietal, right ACC: AD > OA<br>-Task-related encoding v rest magnitude of deactivation in ACC ROI: OA > AD<br>-Task-related recall v completion activation in left PHC, MFG, right superior FG, left parietal: OA > AD<br>-Task-related recall v completion activation in right middle cingulate cortex, precuneus: AD > OA<br>-Task-related recall v rest magnitude of deactivation in right middle cingulate cortex, precuneus ROI: OA > AD<br>-Hyperactivations reflected a failure of deactivation
Vannini et al., 2012[33]	-26 YA (23y)<br>-40 OA (PiB-: 72y, PiB+: 80y)	- PiB DVR in OA in precuneus	-*Design*: adaptation of Sperling et al. (2003) alternating blocks of 2 conditions: face–name pairs (4x3x20 trials)	-Performance in retrieval task:<br>successful hits: YA > PiB+<br>remembered misses: YA > OA<br>-Task-related magnitude of deactivation in the posteromedial cortex in first

	-Cohort: HABS	- PIB+: DVR > 1.6, n = 8 OA	interspersed with visual fixation, cued-recall and 2AFC recognition (4x20)<br>-*Task*: repetitive face–name association encoding, viewing faces on photographs with common names for 2.75 sec (intentional encoding), rating of 'fit', training before<br>-*Contrast*: remembered hit first encoding activation v fixation cross (RHITenc v fixation) (cingulate isthmus, HC ROIs)	encoding trial: YA > OA (right hemisphere), PiB- > PiB+<br>-Decrease of deactivation with repeated encoding trials: YA > PiB- (no decrease in PiB+)<br>-Task-related magnitude of activation in HC in second encoding trial: PiB+ > PiB-<br>-No differences in pattern of decrease of activation in HC over repeated encoding trials between groups
Clément & Belleville, 2012[34]	-14 OA (67y)<br>-26 MCI (higher-cognition: 69y, lower-cognition: 67y, split-median on dementia rating MDRS)<br>-Subjects see Clément & Belleville, 2010	-	-*Design*: see Clément & Belleville, 2010, two types of foils in recognition task: item recognition: old word and new related word as new pair; associative recognition: old word and old word from different pair as rearranged pair<br>-*Task*: verbal learning of word pairs (intentional encoding)<br>-*Contrast*: old/ new v fixation (item recognition; familiarity), intact/ rearranged v fixation (associative recognition; recollection)	-Performance in recognition task: YA/ MCI high-cog. > MCI low-cog.<br>-Task-related extent of activation for associative recognition in left inferior parietal, right temporal and vlPFC, dlPFC: MCI high-cog. > OA<br>-Potential compensation in MCI high-cog.<br>-Task-related extent of activation for item recognition in right PCC, PHC: OA > MCI high-cog.<br>-Task-related extent of activation for item recognition in left dlPFC and inferior parietal, mPFC, ACC,: low-cog. MCI > OA<br>-Task-related extent of activation for associative recognition in precuneus, superior parietal: MCI high-cog. > low-cog. MCI
Kennedy et al., 2012[35]	-137 healthy adults:<br>- 30 YA (30–49y)<br>-46 MA (50-69y)<br>-61 OA (70-89y)<br>-Cohort: Dallas Lifespan Brain Study	-^{18}Florbetapir PET<br>-SUVR precuneus, continuous analysis	-*Design*: alternation of 2 conditions in 3 runs: viewing of pictures (3x32 trials) and fixation (3x32 trials)<br>-*Task*: episodic memory-encoding of natural scenes (incidental encoding) with judgment if water is in image, recognition 20 min after task (no MRI, 96 targets, 96 lures, high/ low confidence recognition rating)<br>-*Contrast*: high confidence remembered trials v fixation	-Performance in recognition task: decrease with age<br>-Greater Aβ burden in precuneus and the following regions is associated with less task activation in frontal inferior temporal gyri after controlling for age<br>-Greater Aβ burden in superior frontal and left middle temporal gyrus is associated with less task deactivation in those regions<br>-Associated with poorer processing speed, verbal fluency, and fluid reasoning in a subgroup of individuals with elevated precuneus Aβ (n = 18, > 60y)<br>-Effect of Aβ mostly equivalent across lifespan<br>-In right superior frontal and left middle temporal region stronger Aβ-activation association in OA
Vannini et al., 2013[36]	-26 YA (23y)<br>-Cohort: HABS<br>-Subjects see Vannini et al., 2012	-	-*Design*: see Vannini et al., 2012<br>-*Task*: repetitive encoding of face–name pairs<br>-*Contrast*: remembered hit first encoding v fixation	-Task-related magnitude of activation in HC ROI:<br>encoding 1 > encoding 2 > encoding 3 (repetition suppression)<br>-Task-related magnitude of deactivation in posteromedial cortex ROI (DMN): encoding 1 > encoding 2 > encoding 3 (repetition enhancement)
Pudas et al., 2013[37]	-45 YA (35y)<br>-51 sOA successful agers (69y)<br>-51 aOA average agers, age-matched)	-	-*Design*: alternating blocks of three conditions: encoding of face-name pairs (6x4 trials), retrieval (6x4 trials), active baseline task (8x4 trials)<br>-*Task*: face–name paired associates (intentional encoding), retrieval: 3AFC for first letter of name, baseline task: simple perceptual discrimination and button-press	-Performance in retrieval task: YA > sOA/ aOA<br>-Task-related magnitude of encoding activation in left HC ROI: YA/ sOA > aOA<br>-Task-related magnitude of encoding activation in left inferior frontal ROI: sOA > YA (otherwise no differences)<br>-Left HC activation correlated with task performance in aOA

			-*Contrast*: encoding v baseline, retrieval v baseline, subsequently recalled items v baseline (significant clusters as ROIs)	
Elman et al., 2014[38]	-22 YA (24y)<br>-OA:<br>16 PiB+ (76y)<br>33 PiB- (77y)<br>-Cohort: BACS	-PiB DVR (global)<br>-PiB+: DVR > 1.07, n = 16 OA	-*Design*: alternation of 2 conditions: scenes (150 trials), fixation<br>-*Task*: scene encoding (incidental encoding) with color photographs of scenes, gist recognition (written statement) with old/new and confidence rating, detail recognition (written statement, 6 trials) with true/ false after 15 min (no MRI)<br>-*Contrast*: encoding for items subsequently remembered during the gist task (hits) v fixation	-Performance in recognition tasks: no group differences<br>-Increase of activation in occipital, superior and medial parietal, inferior temporal cortex with detail memory: YA > PiB-<br>-Increase of activation in occipital, superior and lateral parietal cortex with detail memory: PiB+ > PiB-<br>-Potential compensation in PIB+<br>-Increase of deactivation in angular gyrus, medial parietal cortex with detail memory: PiB- > PiB+<br>-Increase of deactivation in medial parietal cortex with detail memory: PiB- > YA
Huijbers et al., 2014[39]	-21 YA (25y)<br>-48 OA (76y)<br>-Cohort: HABS	-PiB DVR (global and EC)<br>- PiB+: DVR > 1.15, n = 24 OA	-*Design*: 6 runs of 4 alternating conditions: novel face-name pairs (6x25 trials), previously seen face-name pairs (6x20 trials), re-paired face-name pairs (6x5 trials), fixation (6x50 trials)<br>-*Task*: encoding of face-name pairs (intentional encoding), first viewing of ‘previously seen face-name pairs’ and rating of ‘fit’ before scanning, re-paired face-name pairs were studied but presented in a different combination, recognition task (yes/ no) (3x100 trials: 120 old, 30 re-paired, 150 novel, no MRI)<br>-*Contrast*: task induced activation (tasks v fixation) or deactivation (fixation v tasks) (significant clusters as ROIs)	-Performance in recognition task: YA > OA (no differences between PiB+ and PiB-)<br>-Task-related extent of activation in HC: YA > OA<br>-Task-related extent of deactivation in EC: YA > OA<br>-Task-related magnitude of activation in HC ROI: YA > PiB+/-<br>-Task-related magnitude of deactivation in EC ROI: YA > PiB- > PiB+ (strong Aβ effect in encoding hits v fixation)
Bakker et al., 2015[40]	-17 OA (69y)<br>-54 aMCI (20 62.5 mg (71y), 17 125 mg (73y),<br>17 250 mg (71y))<br>-Longitudinal intervention	-	-*Design*: adapted from Yassa et al. (2010) and Bakker et al (2012) double-blind randomized clinical intervention, placebo and levetiracetam aMCI groups (switch for second time of fMRI task), pseudorandomized alternations of three conditions in 8 runs: novel foils (384 trials total), repeated (96 trials total), lures (similar but not identical) (96 trials total)<br>-*Task*: explicit 3AFC pattern separation task, continuous recognition of color photos of objects with judgment: is stimuli new, old or similar to previous one<br>-*Contrast*: lures called similar v novel foils (= correct pattern separation v implicit baseline) (HC ROIs)	-Performance regarding false alarms (lures called old): OA > aMCI<br>-Task-related activation in left DG/ CA3: aMCI > OA (all on placebo)<br><br>-With Levetiracetam:<br>-DG/ CA3 activation in aMCI reduced to level not different from OA: 62.5 mg, 125 mg<br>-Performance regarding false alarms (lures called old) under 62.5 mg and 125 mg:<br>OA = aMCI

Huijbers et al., 2015[41]	-33 MCI CDR 0.5:<br>17 PiB+ (74y)<br>16 PiB- (72y)<br>-Longitudinal<br>-Cohort: Harvard Medical School	-PiB DVR baseline<br>-PiB+: DVR > 1.2, n = 17	-*Design*: see Sperling et al., 2003<br>-*Task*: face-name association encoding (intentional encoding), rating of 'fit' of name to face, 7 longitudinal MRI scans in total over 3y<br>-*Contrast*: novel v repeated (NvR) (significant clusters as ROIs)	-At baseline:<br>-Task-related NvR magnitude of activation in HC ROI: PiB+ > PiB -<br>-HC volume: PiB- > PiB +<br><br>-Longitudinal:<br>-No difference between PiB groups in rate of decrease of task-related HC activation<br>-Rate of HC atrophy, decline on MMSE and CDR: PiB+ > PiB -<br><br>-Baseline NvR activation in HC associated with CDR progression<br>-9 MCI progressed to AD over 3y (7 PiB+)
Oh et al., 2015 [42]	-42 YA (27y)<br>-57 OA (65y):<br>12 Aβ+ (64y)<br>41 Aβ– (65y)	-^{18}F-Flor-betaben Aβ SUVR OA, Aβ+ n = 12	-*Design*: 3 runs of 30 trials, each trial consisting of: 3 sec study, 7 sec retention, 3 sec probe, 3 or 5 sec ITI, 3 conditions in study phase: 1, 3 or 6 items (each 10x per run)<br>-*Task*: letter Sternberg working-memory task with varying load, items are uppercase letters, indication if a presented letter in probe phase was part of study phase<br>-*Contrast*: load 6 v load 3 v load 1 for delay activation (only correct trials)	-Performance (accuracy) in task: no group differences (reaction time: OA > YA)<br>-Brain regions showing parametric increases in activity in relation to WM load (lateral frontal and parietal, medial parietal, inferior temporal): Aβ+ > Aβ- > YA<br>-Brain regions showing parametric decreases in activity in relation to WM load (PCC, left temporoparietal): no group differences
Gordon et al., 2015[43]	-71 OA (63y)<br>-Cohort: Adult Children Study	- PiB MCBP<br>- PiB+: MCBP > 0.23 (25% of OA)<br><br>-CSF: Aβ42, tau, p-tau181	-*Design*: 2 runs of 3 conditions: animacy judgment task followed by two runs of a Stroop task, within each run alteration between task blocks (4x24 trials) and fixation (5 blocks)<br>-*Task*: two attention-demanding cognitive tasks (incidental encoding), animacy task: living and/or nonliving animacy judgment to words, recognition task after Stroop (no MRI, 384 words, half old, half new foils), confidence rating; Stroop task: incongruent, congruent or neutral response<br>-*Contrast*: animate v inanimate, task v fixation, high-conf. hits v misses	-Higher tau and p-tau was associated with response latency for lure trials (slower correct rejection, faster false alarm)<br>-Higher p-tau was associated with higher task block-level activation v fixation of attentional control areas (right dlPFC, ACC) for both tasks<br>-No associations with Aβ
Rieck et al., 2015[44]	-16 YA (33y)<br>-23 high-Aβ OA (77y)<br>-23 matched low-Aβ OA<br>-Cohort: Dallas Lifespan Brain Study	-^{18}F-Florbetapir Aβ SUVR	-*Design*: 2 runs of each 4 blocks of stimuli (4x 8 trials)<br>-*Task*: passive viewing task (grayscale photographs of faces)<br>-*Contrast*: -<br>(analysis of average activation pattern (multivariate barycentric discriminant analysis), neural patterns for 2 independent factors: age, Aβ)	-Task-related activation differences in posterior lingual, inferior temporal gyrus, anterior PHC in YA and Aβ+/Aβ- (age-factor)<br>-Task-related activation in left fusiform gyrus: Aβ+ > Aβ- (independent of age effects)<br>-Decreased fusiform activation related to worse performance in tasks of processing speed in OA (Aβ-factor)
Edelman et al., 2017[45]	-44 OA (76y, 73% female):	-PiB SUVR<br>-Clustering → Aβ+	- *Design*: see Sperling et al., 2003	- No difference in task performance between groups

	-21 PiB+ (77y)<br>-23 PiB- (76y)		-*Task*: face–name association encoding (intentional encoding, 2 repeated familiar pairs, 32 novel pairs), rating of 'fit' of name to face, immediate name-face 2AFC recognition<br><br>-*Contrast*: novel v familiar	- Task-related activation in MTL: PiB+ > PiB- (peak in PHC)<br>- No association between MTL activation and task accuracy in PiB+ group
Marks et al., 2017[46]	-20 YA (23y)<br>-43 OA (79y)<br>-Cohort: BACS	- PiB DVR<br>- PiB+: DVR > 1.07 (45% of OA)<br><br>-FTP SUVR<br>-Braak I/II: n = 23 OA	-*Design*: see Yassa et al., 2010<br>-*Task*: pattern separation task<br>-*Contrast*: lures called similar v novel foils (sCR: subsequent correct rejection), repeated called old v novel foils (sH: sub. hits), lure called old v novel foils (sFA: sub. false alarms) (MTL ROIs)	-Performance regarding false alarms (lures called old):YA > OA<br>-Task-related sH activation in EC, PHC and reduced deactivation in right anterior HC:OA > YA<br>-Task-related sCR activation in HC, right PHC:OA > YA<br>-Task-related sFA activation in PHC and reduced deactivations in anterior HC, right EC:OA > YA<br>-Reduced sH deactivations in OA associated with increased global PiB DVR<br>-OA with increased pathological activation performed worse on the memory paradigm
Leal et al., 2017 [47]	-27 OA (77y)<br>-Longitudinal<br>-Cohort: BACS<br>-Subsample of Mormino et al., 2012	- PiB DVR: 2-3 scans within 3.4y<br>- PIB+: DVR > 1.06, baseline n = 6, end n = 8 OA	-*Design*: see Mormino et al., 2012<br>-*Task*: episodic memory encoding of natural scenes (incidental encoding) with judgment if water is in image,<br>recognition 15 min after task (no MRI)<br>-*Contrast*: high-confidence hits v misses (DM effect, difference due to subsequent memory) (ROI: right HC)	-Right HC activation predicts higher PiB accumulation over time, not seen for control ROI (inferior frontal and occipital gyrus, which showed hyperactivation at baseline in PiB+ like right HC)<br>-No prediction of CVLT memory decline by baseline right HC activation, but by PiB accumulation over time<br>-PiB accumulation over time (but not baseline PiB) mediates relationship between increased right HC activation and memory decline
Tran et al., 2017 [48]	-35 OA matched controls (69y)<br>-42 OA aMCI (*APOE*: 21 E4+: 72y, 21 E4-: 73y)<br>-Subsample of Bakker et al., 2012, 2015 (20 OA and 32 MCI)	-	-*Design*: see Bakker et al., 2012<br>-*Task*: explicit 3AFC pattern separation task, novel foils as baseline<br>-*Contrast*: lures called similar v novel foils (HC ROIs)	-Performance regarding false alarms (lures called old): OA > aMCI (independent of *APOE*)<br>-Task-related activation in left DG/ CA3: aMCI > OA<br>-No difference in activation due to *APOE* status in aMCI
Berron et al., 2018[49]	-43 YA (24y)<br>-44 OA (69y)	-	-*Design*: alternations of 3 conditions in 56 sequences of each 4 stimuli (either 4 objects or 4 scenes): novel (112 trials in total, first 2 per sequence), repeated (56 trials in total), lures (similar but not identical, 56 trials in total), fixation after each trial<br>-*Task*: object- scene mnemonic discrimination task, computer-generated color pictures of indoor objects and empty indoor scenes with judgment task: is stimulus new or old, lures: change in spatial feature, training shortly before	-Performance regarding false alarms (lures called old) for scenes and objects: YA > OA (no difference in hit rate)<br>-Domain specificity for objects in PrC: YA > OA<br>-Less domain specificity in the PrC associated with lower object corrected hit rate in OA<br>-No age-group differences in novelty activation for lures in MTL ROIs for objects or scenes

			-*Contrast*: scene v object, correct lures v repeats (per domain) (HC ROIs, domain-specificity score: subtracting t-values for all object conditions from all scene conditions)	
Reagh et al., 2018[50]	-20 YA (22y)<br>-20 OA (74y)	-	-*Design*: 6 pseudo-randomized blocks with 4 conditions: object targets, object lures, spatial target, spatial lures<br>blocks: study objects in location (6x42 trials) and test spatial or objects (each 3x42 trials, targets and lures),<br>per block 8 trials of a visual perceptual matching task (as baseline)<br>-*Task*: object v spatial mnemonic discrimination task: judgment of color photographs at encoding (indoor/ outdoor) (intentional encoding), judgment at recognition (same (target)/ different (lure), objects: perceptually same or different, spatial: studied objects in same or different location of white screen; lure similarity: high, mild, low<br><br>-*Contrast*: target hits v baseline, target misses v baseline, lure rejections v baseline (MTL ROIs)	-Performance regarding false alarms (lures called old) over all similarity levels for objects:YA > OA (no difference in hit rate)<br>-Lure discrimination for low+middle object similarity: YA > OA<br>-Lure discrimination for middle spatial similarity: YA > OA<br>-Task-related left alEC activation for objects: YA > OA<br>-Higher activation in OA in left alEC is associated with more correct rejections<br>-Task-related left DG/ CA3 activation for objects and spatial: OA > YA (regardless of level of similarity)<br>-Higher activation in OA in DG/ CA3 associated with less correct rejections for objects and spatial
Foster et al., 2018[51]	-42 YA (28y)<br>-62 OA (68y) (high Aβ: 74y), low Aβ: 65y)	-[18]Florbetapir SUVR<br>- high Aβ: SUVR > 1.11, n = 39 OA	-*Design*: alternations of 4 conditions: coordinate judgment (3 difficulty levels), categorical judgment (control condition)<br>-*Task*: spatial distance-judgment task, categorical: dot on left or right side of bar, coordinate: dot closer/ farther away from horizontal bar than length of a vertical reference line which varied over 3 levels of difficulty<br>-*Contrast*: hard coordinate v control (7 cortical ROIs)	-Task performance: YA > OA (lower with higher difficulty in both age groups without interaction)<br>-Task-related activation in angular, middle and superior temporal, mPFC, ACC: slightly high-Aβ > low-Aβ > high-Aβ (quadratic relationship with Aβ, inverted U)<br>-Aβ load moderated effect of activation on task performance in high Aβ group (n=18): SUVR < 1.23: positive association of deactivation and performance, SUVR > 1.66: negative association of deactivation and performance<br>-*APOE*: no significant effect on activation
Oh et al., 2018 [52]	-46 YA (28y)<br>-82 OA (Aβ+: 66y, Aβ-: 65y))	-[18]Flor-betapen SUVR<br>- Aβ+: n = 21 OA	-*Design*: 6 runs of each 2 blocks of 3 conditions: single task (2x12 trials), dual task (2x12 trials), rest (2x same duration as task block)<br>-*Task*: task switching to capture executive control, single task: only one condition, dual task: switching between vowel/ consonant judgment for green letters and lower/ upper-case judgment for red letters, intermixed with response inhibition trials, intellectual activity: composite of education, reading, vocabulary	-Performance in task: no age- or Aβ-group differences<br>-Aβ+: higher intellectual activity associated with increased Aβ deposition and increased task-related activation in lateral and medial frontoparietal cortices (task-negative regions)<br>(for dual v single task)<br>-Potential compensation in Aβ+<br>-Aβ−: higher intellectual activity was associated with lower Aβ deposition and reduced task-related activation in lateral and medial parietal cortex (task-negative regions) (for both task v baseline contrasts)

			-*Contrast*: single task v dual task, single task v rest, dual task v rest	
Jurick et al., 2018[53]	-29 OA (75y)<br>-20 MCI (73y)<br>(12 aMCI; 8 naMCI)<br>-Longitudinal behavioural testing	-	-*Design*: adapted from Sperling et al. (2003), 5 runs of 4 conditions: novel face–name pairs (5x6 trials), repeated once (R1)(5x6 trials), repeated twice (R2)(5x6 trials), fixation<br>-*Task*: face-name associative encoding with stimuli repetition (intentional encoding), rating of 'fit' of name to face, recognition immediately after MRI (3AFC: name 1, name 2, neither) (90 old, 30 foils)<br>-*Contrast*: R1 v R2, novel v R1, novel v R2	-Task performance: rejecting foils and recognising R2 stimuli: OA > MCI<br>-Task-related novel v R2 decrease in activity in PHC, fusiform, cingulate, caudate: OA > MCI<br>-More activity reduction in this ROI with repetition associated with better recognition performance for once repeated stimuli in MCI<br>-More activity reduction in this ROI with repetition associated with higher performance in visual immediate recall in OA<br>-Task-related novel v R1 decrease in activity in left inferior frontal gyrus and left HC: naMCI > aMCI
Sinha et al., 2018[54]	-30 OA (69y) (African American, 15 *APOE4*+, 15 matched E4-)<br>-Cohort: AABHI	-	-*Design*: see Yassa et al., 2010,<br>3 conditions in 1 run: novel foils (192 trials), repeated (96 trials), lures (similar but not identical) (96 trials)<br>-*Task*: explicit 3AFC pattern separation task, continuous recognition of color photos of objects, judgment: is stimuli new, old or similar to previous one<br>-*Contrast*: see Yassa et al., 2010 (MTL ROIs)	-Performance regarding false alarms (lures called old): E4- > E4+<br>-Task-related magnitude of activation during both contrasts in DG/ CA3 and CA1 ROI: E4- > E4+<br>-Higher activation for the retrieval contrast associated with worse discrimination performance
Huijbers et al., 2019[55]	-120 OA (75y)<br>(11 CDR 0.5 but not MCI)<br>-Cohort: HABS	- PiB DVR (120 OA), global/ neocortex (27 PiB+)<br><br>-FTP SUVR (87 OA), ERC and inferior temporal cortex	-*Design*: 6 runs of 3 conditions: novel faces (6x8 trials), famous faces (6x20 trials), fixation (6x28 trials)<br>-*Task*: encoding of novel faces, color photos of unfamiliar individuals and famous faces, judgment if face is known or not, recognition: 48 old targets, 48 novel foils (no MRI)<br>-*Contrast*: hits v misses (activation successful encoding), misses v hits (activation unsuccessful encoding/ deactivation successful encoding) (famous faces not part of analysis)	-Recognition performance: no difference due to Aβ or HC ROI activation<br>-Partial correlation of false alarm rate and inferior temporal tau<br>-Positive encoding success activity (hits v misses) in visual cortex, fusiform gyrus, PHC, HC<br>-Negative encoding success activity (misses v hits) in the posteromedial cortex, ACC, angular gyrus, lateral temporal cortex<br>-No relation between positive encoding success activation in HC ROI and Aβ in cortex or tau accumulation in EC<br>-Inferior temporal tau associated with positive encoding success activation in HC ROI, no interaction with Aβ
Maass et al., 2019[56]	-25 YA (26y)<br>-50 OA (Aβ+: 77y, Aβ-: 79y))<br>-Cohort: BACS	- PiB DVR global<br>-Aβ+: DVR > 1.065, n = 26 OA<br><br>-FTP SUVR Braak III/ IV region<br>-tau+: SUVR > 1.26, n = 18 OA	-*Design*: adapted from Berron et al. (2018), alternation of 3 conditions in 128 sequences of each 4 stimuli (either 4 objects or 4 scenes): novel (256 trials in total, first 2 per sequence), repeated (128 trials in total), lures (similar but not identical) (128 trials in total) and scrambled noise images (40 trials in total, perceptual baseline)<br>-*Task*: object- scene mnemonic discrimination task, computer-generated color pictures of indoor objects and empty indoor scenes, lures: change in	-Task performance for repeat and lure: YA > OA<br>-Task performance for object lures (relative to scene lures): tau- > tau+<br>-Task-related object activation in PM: tau+ > tau-<br>-Domain-specific activation in PM: tau- > tau+<br>-Higher domain-specific activation in PM in OA associated with higher corrected hit rate (objects and scenes)<br>-Task-related object and scene activation in AT and HC: tau+ > tau-<br>-No differences in activation due to Aβ, no interaction tau and Aβ<br>-Best model to predict PM tau: activation in PM regions and global Aβ as independent predictors

			spatial feature, training shortly before, judgment: is stimulus new or old<br>-*Contrast*: object/ scene v perceptual baseline, object v scene (AT and PM ROIs)	
Nyberg et al., 2019[57]	-21 OA remainers<br>-33 OA dropouts (baseline: 77y)<br>-Longitudinal<br>-Cohort: Betula	-	-*Design*: see Pudas et al., 2013,<br>baseline and follow-up after 4y<br>-*Task*: face-name paired-associates task<br>-*Contrast*: encoding v control, retrieval v control (HC, PFC ROIs)	-Task performance at baseline: remainers > dropouts<br>-Cross-sectional and longitudinal encoding-related hypoactivation in aHC with age (baseline (n = 323) + follow-up (n = 186))<br>-Remainers v dropouts:<br>-Encoding-related hyperactivation right aHC, pHC, PFC: dropouts > remainers<br>-Encoding-related hyperconnectivity right PFC - aHC: dropouts > remainers
Corriveau-Lecavalier et al., 2019[58]	-14 OA (67y)<br>-26 MCI (68y, progression to AD: 13))<br>-Longitudinal<br>-Subjects see Clément & Belleville, 2010	-	-*Design*: see Clément & Belleville, 2010, baseline and follow-up after 2y<br>-*Task*: verbal learning of word pairs (intentional encoding)<br>-*Contrast*: encoding v fixation (ROIs: where greater cortical thinning in MCI > OA and HC ROI)	-Task performance ((hit rates/total stimuli) – (false alarm/total stimuli)): OA > all MCI<br>-Task-related activation in right supramarginal gyrus ROI: pMCI > OA<br>-Task-related activation in left opercularis ROI: OA > MCI<br>-Task-related activation in left HC ROI: OA > MCI<br>-All no group x time interaction<br>-No correlation between activation and time to dementia
Corona-Long et al., 2020[59]	-35 OA (18m: 70y, 17f: 68y)<br>-42 aMCI (CDR 0.5) (19m: 71y, 23f: 72y)<br>-Subsample (20 OA and 32 MCI) of Bakker et al., 2012, 2015; sample of Tran et al., 2017	-	-*Design*: see Bakker et al., 2012<br>-*Task*: explicit 3AFC pattern separation task, novel foils as baseline<br>-*Contrast*: lures called similar v novel foils (HC ROIs)	-Task performance: no differences due to sex<br>-Performance regarding false alarms (lures called old): OA > aMCI<br>-Task-related activation in DG/ CA3: no differences due to sex<br>-Task-related activation in DG/ CA3: aMCI > OA
Adams et al., 2021[60]	-21 YA (27y)<br>-45 OA (78y)<br>-Cohort: BACS<br>-Subsample of Maass et al., 2019	- PiB DVR global<br>-Aβ+: DVR > 1.065, n = 23 OA<br><br>-FTP SUVR Braak III/ IV region<br>-tau+: SUVR > 1.26, n = 16 OA	-*Design*: see Maass et al., 2019<br>-*Task*: object- scene mnemonic discrimination task,<br>focus on repetition suppression, collapsing objects and scenes<br>-*Contrast*: novel v baseline, repeats v baseline, novel v repeats (MTL ROIs)	-Task performance: no association with MTL repetition suppression or EC tau deposition<br>-Repetition suppression over all MTL ROIs:<br>YA > OA tau- > OA tau+<br>-Repetition suppression in HC and right alEC: YA > OA tau-<br>-Repetition suppression in left HC, left PHC, right pmEC, right BA35, BA36: OA tau- > OA tau+<br>-Less repetition suppression driven by hyperactivity to repeated stimuli, not less activation for novelty<br>- No differences between PiB- and PiB+<br>-Higher EC tau associated with greater activation in BA35, pmEC and HC for both novel and repeats, but no association with global PiB or interaction with PiB status
Corriveau-Lecavalier et al., 2020[61]	-54 0A (72y)<br>-28 SCD$^+$ (73y) (smaller HC volumes and/or *APOE4*)<br>-26 MCI (76y)<br>-Cohort: CIMA-Q	-	- *Design*: alternation of objects (78 trials) and gray squares (39 trials) in one of the 4 quadrants of the screen<br>-*Task*: object-location associative encoding task: encoding of pictures of concrete items as well as their emplacement in a four-position grid	-Task performance (memory score: correct source/ (wrong source + false alarm)): OA > MCI (no difference between SCD$^+$ and OA or MCI)<br>-Quadratic association between cortical thickness in AD signature regions/ associative memory performance and activation in left superior parietal lobule in SCD$^+$/ MCI

			(intentional encoding), gray squares as control condition, button-press when item visible, recognition 10 min after scan (78 old targets, 39 novel foils) (no MRI), recall of location if recognised<br>-*Contrast*: successful item and location memory v control<br>(HC, AD-related cortical ROIs)	-Linear negative association of left HC volume and left HC/ right inferior temporal activation<br>-Task-related activation in HC, middle temporal, left superior parietal, right inferior temporal lobe, right precuneus: SCD$^+$ > OA/ MCI
Corriveau-Lecavalier et al., 2021[62]	-26 OA (71y)<br>-28 SCD$^+$ (73y) -26 MCI (76y)<br>-Cohort: CIMA-Q<br>-Subjects see Corriveau-Lecavalier et al., 2020	-	*Design*: see Corriveau-Lecavalier et al., 2020<br>-*Task*: object-location associative encoding task (intentional encoding)<br>-*Contrast*: -<br>(HC, AD-related cortical ROIs)(successful item and location memory, seed-partial least square analyses (seed-PLS) for latent variables of task-activation functional networks)	-Task performance (memory score): no differences between groups<br>-Higher HC activation and fronto-temporo-cerebellar connectivity relate to worst memory performance in SCD$^+$ and MCI<br>-Higher right inferior temporal activation and stronger connectivity within fronto-temporal network associated with better memory performance in SCD$^+$ and MCI<br>-In SCD$^+$ also for left superior parietal activation<br>-Potential compensation in SCD$^+$ and MCI
Diersch et al., 2021[63]	- Behavior:<br>17 YA (24y)<br>17 OA (66y)<br>-fMRI:<br>25 YA (23y)<br>32 OA (67y)	-	-*Design*: alternation of 4 conditions in 8 blocks: encoding (8x7 trials, first encoding was familiarization outside scanner), retrieval (8x8 trials), control (8x4 trials), fixation<br>-*Task*: spatial learning task, photorealistic virtual environment of a city center, retrieval: pointing in direction, control trials: indicate change in color, training for pointing before<br>-*Contrast*: retrieval v control, travel during retrieval v travel same area during encoding<br>(Bayesian state-space model for learning states, ROIs: HC, RSC/ POS, DCM PEB)	-Task performance (correct pointing): YA > OA<br>-Learning in both groups, yet YA stronger improvement and ceiling performance after first 2 leaning blocks<br>-Task-related retrieval v control activation in left POS, left PSC, left aHC: OA > YA<br>-Task-related retrieval v control activation in right POS: YA > OA<br>-Task-related retrieval v encoding activation in right POS, aHC: OA > YA<br>-Learning-related decrease in HC activation and increase in RSC/ POS activation: YA > OA<br>-DCM HC-POS: learning deficits in OA linked to increase in aHC excitability (reduction inhibition self-connection)
Soch et al., 2021 [64]	-106 YA (24y)<br>-111 OA (67y)<br>-Cohort: young subjects: DELCODE	-	-*Design*: alternation of 3 conditions: novel scenes (88 trials), familiar scenes (22 repetitions of each 2 scenes), fixation<br>-*Task*: scene encoding task (incidental encoding), indoor/outdoor judgment of scenes<br>-*Contrast*: items later remembered v later forgotten	-Task-related activation in left PHC, right dlPFC: YA > OA<br>-Task-related deactivation in precuneus, PCC, pACC: YA > OA
Billette et al., 2022[65]	-163 OA (70y)<br>-222 SCD (70y)<br>-82 MCI (74y)<br>-32 mild AD (74y)<br>-Cohort: DELCODE	-	-*Design*: alternation of 3 conditions: novel scenes (88 trials), familiar scenes (22 repetitions of each 2 scenes), fixation<br>-*Task*: scene encoding task (incidental encoding), indoor/outdoor judgment of scenes<br>-*Contrast*: novel v highly familiar scenes<br>(ROIs: EC, HC, precuneus)	-Task-related activation in precuneus: MCI > OA/ AD; SCD > OA (inverted U)<br>-Quadratic relationship (inverted U) between memory impairment and precuneus activation (more impairment associated with higher activation in OA/ SCD and lower activation in AD)<br>-Task-related activation in HC: OA > AD<br>-Differences in activation not related to CSF Aβ, p-tau, brain volume (VBM) or *APOE4* genotype

Adams et al., 2022[66]	-37 OA (77y)<br>-Longitudinal<br>-Cohort: BACS	- PiB DVR global<br>- Aβ+: DVR > 1.065, n = 17 OA<br><br>-FTP SUVR MTL	-*Design*: see Maass et al., 2019<br>-*Task*: object- scene mnemonic discrimination task<br>-*Contrast*: all stimuli v perceptual baseline<br>(MTL ROIs)	-No association between activation and tau at baseline<br>-Higher baseline activation in EC and HC associated with more longitudinal EC tau<br>-Higher PHC baseline activation associated with more longitudinal PHC tau<br>-Higher baseline IT activation associated with more global Aβ accumulation<br>-No effect of *APOE* on tau-slopes
Corriveau-Lecavalier et al., 2022[67]	- male:<br>11 OA (70y)<br>32 SCD (73y)<br>18 MCI (75y)<br>-female:<br>24 OA (73y)<br>68 SCD (72y)<br>29 MCI (76y)<br>-Cohort: CIMA-Q	-	*Design*: see Corriveau-Lecavalier et al., 2020<br>-*Task*: object-location associative encoding task (intentional encoding)<br>-*Contrast*: -<br>(successful item and location memory for various male v female contrasts)<br>(multivariate non-rotated behavioral partial least squares correlation (PLSC) analysis with whole-brain activation and associative memory score)	-No sex difference between male and female OA<br>- Overall sex differences across diagnoses:<br>lateral inferior and middle frontal, inferior parietal, and ventral occipito-temporal cortex, PHC, thalamus<br>-Successful encoding activation there correlated positively with memory performance in males, negatively in females<br>-Successful encoding activation in dmFC, PCC, precuneus correlated positively with memory performance in SCD males, not SCD females<br>-Patterns of hyperactivation related to worse memory in females and better memory in males<br>-Potential compensation in males
Kizilirmak et al., 2023[68]	-106 YA (24y)<br>-111 OA (67y)<br>-Cohort: young subjects: DELCODE<br>-Subjects see Soch et al., 2021	-	-*Design*: alternation of 3 conditions: novel scenes (88 trials), familiar scenes (22 repetitions of each 2 scenes), fixation<br>-*Task*: scene encoding task (incidental encoding), indoor/outdoor judgment of scenes<br>-*Contrast*: items later remembered v later forgotten (subsequent memory effect (SME))<br>(ROIs: DMN, mPFC, ACC, PCC, precuneus; resting-state measure mPerAF)	-Task-related deactivations and resting-state amplitudes in precuneus, pACC, AG: YA > OA<br>-OA with higher DMN mPerAF show higher task memory performance<br>-OA with higher task-related activation in precuneus show worse performance in delayed verbal recall task
Giorgio et al., 2023[69]	-21 YA (27y)<br>-45 OA (78y)<br>-Longitudinal tau PET<br>-Cohort: BACS	-PET DVR global<br>-Aβ+: DVR > 1.065, N = 21 OA<br><br>-FTP SUVR EC	-*Design*: see Maass et al., 2019<br>-*Task*: object- scene mnemonic discrimination task<br>-*Contrast*: objects v scenes, novel v repeated<br>(ICA-derived cortical functional networks, DCM PEB)	-Progressive hyperexcitation in DMN with increasing levels of Aβ, which in turn drives hyperexcitability within MTL<br>-Directed hyperexcitation of MTL by DMN pre-empts rate of tau accumulation within EC (longitudinal)

This table highlights methodological details and key findings from the task-based fMRI studies across the aging and Alzheimer's disease cascade cited in this review. Consolidating methodological information such as sample type and size, biomarkers assessed, specific task designs and contrasts analyzed into a single table will help with interpretation of seemingly disparate findings from the literature, and set the foundation for the model depicted in Figure 4.
OA = older adults; APOE = apolipoprotein E; HC = hippocampus; PHC = parahippocampal cortex; PFC = prefrontal cortex; ACC = anterior cingulate cortex; y = years; YA = young adults; AD = Alzheimer's disease patients; MRI = magnetic resonance imaging; MCI = mild cognitive impairment patients; min = minutes; sec = second; PCC = posterior cingulate cortex; MTL = medial temporal lobe; VBM = voxel-based morphometry; EC = entorhinal cortex; CVLT = California Verbal Learning Test; 2AFC = two-alternative forced choice; MFC = medial frontal cortex; GMV = gray matter volume; SCI = subjective cognitive impairment subjects; DMN = default mode network; PiB = Pittsburgh Compound B; DVR = distribution volume; CDR = Clinical Dementia Rating; aMCI = amnestic MCI; RAVLT = Rey Auditory Verbal Learning Test; DG/ CA3 = dentate gyrus/ CA3; vl = ventrolateral; dl = dorsolateral; mPFC = medial prefrontal cortex; MFG = middle frontal gyrus; SUVR = standard uptake volume ratio; Aβ = amyloid beta; MMSE = Mini-Mental State Examination; WM = working memory; MCBP = mean cortical binding potential; FTP = Flortaucipir; PM = posterior midline regions, AT = anterior-temporal regions; aHC = anterior hippocampus; pHC = posterior hippocampus; RSC = retrosplenial cortex; POS = parieto-occipital sulcus; pACC = pregenual anterior cingulate cortex. statistical terms are not listed.

References

1. Bookheimer SY, Strojwas MH, Cohen MS, et al. Patterns of brain activation in people at risk for Alzheimer's disease. *N Engl J Med*. 2000;343(7):450-456.
2. Lustig C, Snyder AZ, Bhakta M, et al. Functional deactivations: change with age and dementia of the Alzheimer type. *Proc Natl Acad Sci U S A*. 2003;100(24):14504-14509.
3. Machulda MM, Ward HA, Borowski B, et al. Comparison of memory fMRI response among normal, MCI, and Alzheimer's patients. *Neurology*. 2003;61(4):500-506.
4. Sperling RA, Bates JF, Chua EF, et al. fMRI studies of associative encoding in young and elderly controls and mild Alzheimer's disease. *J Neurol Neurosurg Psychiatry*. 2003;74(1):44-50.
5. Johnson SC, Schmitz TW, Moritz CH, et al. Activation of brain regions vulnerable to Alzheimer's disease: The effect of mild cognitive impairment. *Neurobiol Aging*. 2006;27(11):1604-1612.
6. Greicius MD, Srivastava G, Reiss AL, Menon V. Default-mode network activity distinguishes Alzheimer's disease from healthy aging: evidence from functional MRI. *Proc Natl Acad Sci U S A*. 2004;101(13):4637-4642.
7. Dickerson BC, Salat DH, Bates JF, et al. Medial temporal lobe function and structure in mild cognitive impairment. *Ann Neurol*. 2004;56(1):27-35.
8. Bondi MW, Houston WS, Eyler LT, Brown GG. fMRI evidence of compensatory mechanisms in older adults at genetic risk for Alzheimer disease. *Neurology*. 2005;64(3):501-508.
9. Dickerson BC, Salat DH, Greve DN, et al. Increased hippocampal activation in mild cognitive impairment compared to normal aging and AD. *Neurology*. 2005;65(3):404-411.
10. Celone KA, Calhoun VD, Dickerson BC, et al. Alterations in Memory Networks in Mild Cognitive Impairment and Alzheimer's Disease: An Independent Component Analysis. *J Neurosci*. 2006;26(40):10222-10231.
11. Kircher TT, Weis S, Freymann K, et al. Hippocampal activation in patients with mild cognitive impairment is necessary for successful memory encoding. *J Neurol Neurosurg Psychiatry*. 2007;78(8):812-818.
12. Wright CI, Dickerson BC, Feczko E, Negeira A, Williams D. A functional magnetic resonance imaging study of amygdala responses to human faces in aging and mild Alzheimer's disease. *Biol Psychiatry*. 2007;62(12):1388-1395.
13. Hämäläinen A, Pihlajamäki M, Tanila H, et al. Increased fMRI responses during encoding in mild cognitive impairment. *Neurobiol Aging*. 2007;28(12):1889-1903.
14. Miller SL, Celone K, DePeau K, et al. Age-related memory impairment associated with loss of parietal deactivation but preserved hippocampal activation. *Proc Natl Acad Sci U S A*. 2008;105(6):2181-2186.
15. Miller SL, Fenstermacher E, Bates J, Blacker D, Sperling RA, Dickerson BC. Hippocampal activation in adults with mild cognitive impairment predicts subsequent cognitive decline. *J Neurol Neurosurg Psychiatry*. 2008;79(6):630-635.
16. Pihlajamäki M, DePeau KM, Blacker D, Sperling RA. Impaired medial temporal repetition suppression is related to failure of parietal deactivation in Alzheimer disease. *Am J Geriatr Psychiatry*. 2008;16(4):283-292.
17. Rodda JE, Dannhauser TM, Cutinha DJ, Shergill SS, Walker Z. Subjective cognitive impairment: increased prefrontal cortex activation compared to controls during an encoding task. *Int J Geriatr Psychiatry*. 2009;24(8):865-874.
18. Sperling RA, Laviolette PS, O'Keefe K, et al. Amyloid deposition is associated with impaired default network function in older persons without dementia. *Neuron*. 2009;63(2):178-188.
19. Filippini N, MacIntosh BJ, Hough MG, et al. Distinct patterns of brain activity in young carriers of the APOE-epsilon4 allele. *Proc Natl Acad Sci U S A*. 2009;106(17):7209-7214.
20. Dennis NA, Browndyke JN, Stokes J, et al. Temporal lobe functional activity and

connectivity in young adult APOE ε4 carriers. *Alzheimers Dement*. 2010;6(4):303-311.
21. Quiroz YT, Budson AE, Celone K, et al. Hippocampal hyperactivation in presymptomatic familial Alzheimer's disease. *Ann Neurol*. 2010;68(6):865-875.
22. O'Brien JL, O'Keefe KM, LaViolette PS, et al. Longitudinal fMRI in elderly reveals loss of hippocampal activation with clinical decline. *Neurology*. 2010;74(24):1969-1976.
23. Clément F, Belleville S. Compensation and Disease Severity on the Memory-Related Activations in Mild Cognitive Impairment. *Biol Psychiatry*. 2010;68(10):894-902.
24. Yassa MA, Muftuler LT, Stark CEL. Ultrahigh-resolution microstructural diffusion tensor imaging reveals perforant path degradation in aged humans in vivo. *Proc Natl Acad Sci U S A*. 2010;107(28):12687-12691.
25. Pihlajamäki M, O'Keefe K, O'Brien J, Blacker D, Sperling RA. Failure of repetition suppression and memory encoding in aging and Alzheimer's disease. *Brain Imaging Behav*. 2011;5(1):36-44.
26. Putcha D, Brickhouse M, O'Keefe K, et al. Hippocampal Hyperactivation Associated with Cortical Thinning in Alzheimer's Disease Signature Regions in Non-Demented Elderly Adults. *J Neurosci*. 2011;31(48):17680-17688.
27. Erk S, Spottke A, Meisen A, Wagner M, Walter H, Jessen F. Evidence of Neuronal Compensation During Episodic Memory in Subjective Memory Impairment. *Arch Gen Psychiatry*. 2011;68(8):845-852.
28. Rodda J, Dannhauser T, Cutinha DJ, Shergill SS, Walker Z. Subjective cognitive impairment: functional MRI during a divided attention task. *Eur Psychiatry*. 2011;26(7):457-462.
29. Yassa MA, Lacy JW, Stark SM, Albert MS, Gallagher M, Stark CEL. Pattern separation deficits associated with increased hippocampal CA3 and dentate gyrus activity in nondemented older adults. *Hippocampus*. 2011;21(9):968-979.
30. Bakker A, Krauss GL, Albert MS, et al. Reduction of hippocampal hyperactivity improves cognition in amnestic mild cognitive impairment. *Neuron*. 2012;74(3):467-474.
31. Mormino EC, Brandel MG, Madison CM, Marks S, Baker SL, Jagust WJ. Aβ Deposition in aging is associated with increases in brain activation during successful memory encoding. *Cereb Cortex*. 2012;22(8):1813-1823.
32. Bejanin A, Viard A, Chételat G, et al. When Higher Activations Reflect Lower Deactivations: A PET Study in Alzheimer's Disease during Encoding and Retrieval in Episodic Memory. *Front Hum Neurosci*. 2012;6:107.
33. Vannini P, Hedden T, Becker JA, et al. Age and amyloid-related alterations in default network habituation to stimulus repetition. *Neurobiol Aging*. 2012;33(7):1237-1252.
34. Clément F, Belleville S. Effect of Disease Severity on Neural Compensation of Item and Associative Recognition in Mild Cognitive Impairment. *J Alzheimers Dis*. 2012;29(1):109-123.
35. Kennedy KM, Rodrigue KM, Devous MD Sr, Hebrank AC, Bischof GN, Park DC. Effects of beta-amyloid accumulation on neural function during encoding across the adult lifespan. *Neuroimage*. 2012;62(1):1-8.
36. Vannini P, Hedden T, Sullivan C, Sperling RA. Differential functional response in the posteromedial cortices and hippocampus to stimulus repetition during successful memory encoding. *Hum Brain Mapp*. 2013;34(7):1568-1578.
37. Pudas S, Persson J, Josefsson M, de Luna X, Nilsson LG, Nyberg L. Brain characteristics of individuals resisting age-related cognitive decline over two decades. *J Neurosci*. 2013;33(20):8668-8677.
38. Elman JA, Oh H, Madison CM, et al. Neural compensation in older people with brain amyloid-β deposition. *Nat Neurosci*. 2014;17(10):1316-1318.
39. Huijbers W, Mormino EC, Wigman SE, et al. Amyloid deposition is linked to aberrant entorhinal activity among cognitively normal older adults. *J Neurosci*. 2014;34(15):5200-5210.
40. Bakker A, Albert MS, Krauss G, Speck CL, Gallagher M. Response of the medial temporal lobe network in amnestic mild cognitive impairment to therapeutic intervention assessed by fMRI and memory task performance. *NeuroImage: Clinical*.

2015;7:688-698.
41. Huijbers W, Mormino EC, Schultz AP, et al. Amyloid-β deposition in mild cognitive impairment is associated with increased hippocampal activity, atrophy and clinical progression. *Brain*. 2015;138(Pt 4):1023-1035.
42. Oh H, Steffener J, Razlighi QR, et al. Aβ-related hyperactivation in frontoparietal control regions in cognitively normal elderly. *Neurobiol Aging*. 2015;36(12):3247-3254.
43. Gordon BA, Zacks JM, Blazey T, et al. Task-evoked fMRI changes in attention networks are associated with preclinical Alzheimer's disease biomarkers. *Neurobiol Aging*. 2015;36(5):1771-1779.
44. Rieck JR, Rodrigue KM, Kennedy KM, Devous MD Sr, Park DC. The effect of beta-amyloid on face processing in young and old adults: A multivariate analysis of the BOLD signal. *Hum Brain Mapp*. 2015;36(7):2514-2526.
45. Edelman K, Tudorascu D, Agudelo C, et al. Amyloid-Beta Deposition is Associated with Increased Medial Temporal Lobe Activation during Memory Encoding in the Cognitively Normal Elderly. *Am J Geriatr Psychiatry*. 2017;25(5):551-560.
46. Marks SM, Lockhart SN, Baker SL, Jagust WJ. Tau and β-Amyloid Are Associated with Medial Temporal Lobe Structure, Function, and Memory Encoding in Normal Aging. *J Neurosci*. 2017;37(12):3192-3201.
47. Leal SL, Landau SM, Bell RK, Jagust WJ. Hippocampal activation is associated with longitudinal amyloid accumulation and cognitive decline. Published online February 8, 2017. doi:10.7554/eLife.22978
48. Tran TT, Speck CL, Pisupati A, Gallagher M, & Bakker A. Increased hippocampal activation in ApoE-4 carriers and non-carriers with amnestic mild cognitive impairment. *NeuroImage: Clinical*. 2017;13:237-245.
49. Berron D, Neumann K, Maass A, et al. Age-related functional changes in domain-specific medial temporal lobe pathways. *Neurobiol Aging*. 2018;65:86-97.
50. Reagh ZM, Noche JA, Tustison NJ, Delisle D, Murray EA, Yassa MA. Functional Imbalance of Anterolateral Entorhinal Cortex and Hippocampal Dentate/CA3 Underlies Age-Related Object Pattern Separation Deficits. *Neuron*. 2018;97(5):1187-1198.e4.
51. Foster CM, Kennedy KM, Horn MM, Hoagey DA, Rodrigue KM. Both hyper- and hypo-activation to cognitive challenge are associated with increased beta-amyloid deposition in healthy aging: A nonlinear effect. *Neuroimage*. 2018;166:285-292.
52. Oh H, Razlighi QR, Stern Y. Multiple pathways of reserve simultaneously present in cognitively normal older adults. *Neurology*. 2018;90(3):e197.
53. Jurick SM, Weissberger GH, Clark LR, et al. Faulty Adaptation to Repeated Face-Name Associative Pairs in Mild Cognitive Impairment is Predictive of Cognitive Decline. *Arch Clin Neuropsychol*. 2018;33(2):168-183.
54. Sinha N, Berg CN, Tustison NJ, et al. APOE ε4 status in healthy older African Americans is associated with deficits in pattern separation and hippocampal hyperactivation. *Neurobiology of Aging*. 2018;69:221-229.
55. Huijbers W, Schultz AP, Papp KV, et al. Tau Accumulation in Clinically Normal Older Adults Is Associated with Hippocampal Hyperactivity. *J Neurosci*. 2019;39(3):548-556.
56. Maass A, Berron D, Harrison TM, et al. Alzheimer's pathology targets distinct memory networks in the ageing brain. *Brain*. 2019;142(8):2492-2509.
57. Nyberg L, Andersson M, Lundquist A, Salami A, Wåhlin A. Frontal Contribution to Hippocampal Hyperactivity During Memory Encoding in Aging. *Front Mol Neurosci*. 2019;12:229.
58. Corriveau-Lecavalier N, Mellah S, Clément F, Belleville S. Evidence of parietal hyperactivation in individuals with mild cognitive impairment who progressed to dementia: A longitudinal fMRI study. *NeuroImage: Clinical*. 2019;24:101958.
59. Corona-Long CA, Tran TT, Chang E, Speck CL, Gallagher M, Bakker A. Comparison of male and female patients with amnestic mild cognitive impairment: Hippocampal hyperactivity and pattern separation memory performance. *Alzheimers Dement*. 2020;12(1):e12043.
60. Adams JN, Maass A, Berron D. Reduced Repetition Suppression in Aging is Driven by Tau–Related Hyperactivity in Medial Temporal Lobe. *Journal of*. Published online

2021. https://www.jneurosci.org/content/41/17/3917.abstract
61. Corriveau-Lecavalier N, Duchesne S, Gauthier S, et al. A quadratic function of activation in individuals at risk of Alzheimer's disease. *Alzheimer's & Dementia: Diagnosis, Assessment & Disease Monitoring*. 2020;12(1):e12139.
62. Corriveau-Lecavalier N, Rajah MN, Mellah S, Belleville S. Latent patterns of task-related functional connectivity in relation to regions of hyperactivation in individuals at risk of Alzheimer's disease. *NeuroImage: Clinical*. 2021;30:102643.
63. Diersch N, Valdes-Herrera JP, Tempelmann C, Wolbers T. Increased Hippocampal Excitability and Altered Learning Dynamics Mediate Cognitive Mapping Deficits in Human Aging. *J Neurosci*. 2021;41(14):3204-3221.
64. Soch J, Richter A, Schütze H, et al. A comprehensive score reflecting memory-related fMRI activations and deactivations as potential biomarker for neurocognitive aging. *Hum Brain Mapp*. 2021;42(14):4478-4496.
65. Billette OV, Ziegler G, Aruci M, et al. Novelty-Related fMRI Responses of Precuneus and Medial Temporal Regions in Individuals at Risk for Alzheimer Disease. *Neurology*. 2022;99(8):e775-e788.
66. Adams JN, Kim S, Rizvi B, et al. Entorhinal-Hippocampal Circuit Integrity Is Related to Mnemonic Discrimination and Amyloid-β Pathology in Older Adults. *J Neurosci*. 2022;42(46):8742-8753.
67. Corriveau-Lecavalier N, Décarie-Labbé L, Mellah S, Belleville S, Rajah MN, Consortium for the Early Identification of Alzheimer's Disease-Quebec (CIMA-Q). Sex differences in patterns of associative memory-related activation in individuals at risk of Alzheimer's disease. *Neurobiol Aging*. 2022;119:89-101.
68. Kizilirmak JM, Soch J, Richter A, Schott BH. Age-related differences in fMRI subsequent memory effects are directly linked to local grey matter volume differences. *bioRxiv*. Published online February 23, 2023:2023.02.23.529668. doi:10.1101/2023.02.23.529668
69. Giorgio J, Adams JN, Maass A, Jagust W, Breakspear M. Amyloid Induced Hyperexcitability in Default Mode Network Drives Medial Temporal Hyperactivity and Early Tau Accumulation. *Neuron*. Published online January 1, 2023. doi:10.2139/ssrn.4404292
